# Sexual reproduction in plagiogrammacean diatoms: First insights into the early pennates

**DOI:** 10.1371/journal.pone.0181413

**Published:** 2017-08-16

**Authors:** Irena Kaczmarska, Benjamin S. Gray, James M. Ehrman, Mary Thaler

**Affiliations:** 1 Department of Biology, Mount Allison University, Sackville, New Brunswick, Canada; 2 Digital Microscopy Facility, Mount Allison University, Sackville, New Brunswick, Canada; 3 Institut de biologie intégrative et des systèmes, Université Laval, Québec, Québec, Canada; University of Connecticut, UNITED STATES

## Abstract

The genera *Plagiogramma* and *Dimeregramma* are members of a small, but evolutionarily important group of diatoms, the "basal" araphids. They are sister to all other pennates, both araphid and raphid taxa. Thus, their phylogenetic position carries the potential for providing insights into the earliest pennates. We documented sexual reproduction, mating system and sex cell development in the first members of the "basal" araphid clade ever investigated. The mating system in all these species involved heterothally. It was, however, more complex in *P*. *tsawwassen*, where in addition to heterothallic clones, intraclonal and polysexual clones also exist. Auxospore development and wall structure was similar in all three species and demonstrated several characters also reported from "core" araphids. Of these, vigorous, pseudopodial motility of male secondary spermatocytes and gametes was most notable because it indicates that this character was likely present in the last common ancestor of all the pennates. Pseudopodial motility of the male sex cells might have afforded sufficient compensation and/or benefits to the emerging pennates for replacing flagellated sperm, present in centrics. The characters thus far uniquely present among our plagiogrammaceans but not reported from other pennates were: the "gametic" fusion between sex-compatible secondary spermatocytes, in some cases before completion of Meiosis II in males, transverse perizonial bands produced all together or in quick succession rather than being added to the auxospore apex one at a time, and expanding auxospores with 3–4 nuclei. An initial epivalve, similar in morphology to what in some diatoms had been interpreted as a “longitudinal” perizonium, may be more widespread among pennates than thus far appreciated. In addition, we discovered two species new to science (*D*. *acutumontgo*, *P*. *tsawwassen*), and refined delineation of *P*. *staurophorum* by including metric data from the original material.

## Introduction

Modern research interest in diatom auxospore structure and development began as soon as electron microscopes became commercially available; first using transmission electron microscopy (TEM) in the early 1970s [1, 2], and then scanning electron microscopy (SEM) about a decade later [3, 4]. This work delivered many unanticipated results, some of which culminated in novel ideas about the origin of diatoms [5, 6] and relationships between various lineages within diatoms [2, 7, 8]. Most of that research, however, focused on centrics and raphid pennates. Marine araphid diatoms, on the other hand, are one of the least understood groups of diatoms in terms of their reproductive strategies, structure of the cells involved, and their behaviour. Because their occurrence, mostly in benthic habitats, is unpredictable and seldom at high abundances, these species are infrequently investigated using combined morphological and molecular approaches. Even fewer targeted studies used combined morphological and molecular methods to examine sexually compatible clones. Compatibility studies will likely be necessary to disentangle some of the most notoriously difficult to identify (and delineate) fragillarioid and synedrid species, cases of likely similar complexity to cryptic and semi-cryptic members of the genera *Pseudo-nitzschia* or *Asterionellopsis* [9, 10].

To date, sexual reproduction has been studied for species in only about a dozen araphid genera, e.g., [11–16]. From these and earlier works [2, 17–19], it has become evident that araphid pennates evolved some very unusual types of sex cell and auxospore structures that are not shared with centric or pennate diatoms. For example, a unique form of oogamy is known in species of the genus *Rhabdonema* [18], and non-flagellated but vigorously motile male gametes in *Tabularia*, *Pseudostaurosira*, and *Ulnaria* [20–23], among other characters. This leads to the question of whether any of these unusual characters were present in the earliest of the araphid pennates, or if they are restricted to the more recently diverged members. Our understanding of the evolution of pennates from among the polar centric pool, and their own subsequent diversification, depends on the answers to such questions.

There is a long-standing consensus that araphid pennates emerged from among the polar centrics, and that raphid diatoms diverged from araphid ancestors [24–26]. However, which araphid diatom lineages share the most recent common ancestry with which polar centrics and which of them with the raphid pennates remains to be established. Although the evidence from araphid valve structure [27] suggests that araphid diatoms are a coherent entity based on the presence of labiate processes (and the absence of a raphe), several different molecular phylogenies suggest that araphid diatoms are not monophyletic. Instead, the group consists of the so-called "basal araphids", sister to all remaining raphid and araphid pennates, and the "core araphids" which are sister to raphid pennate diatoms [28–32]. Irrespective of the study, most agree that one of the earliest pennate divergences contains rhaphoneidacean, asterionellopsid and plagiogrammacean diatoms (when all are included in the analysis; [26, 29, 32–35]). The three groups tend to join either all together as a sister clade to all other pennates, or emerge one after another at the base of the pennates. In this light, it is apparent that all the modern studies of diatom sexuality and reproductive structures to date include only the members of more divergent, “core” araphid families (Fragillariaceae, Tabulariaceae, Rhabdonemaceae, Licmophoraceae *sensu* reference [27], etc.; [13–15, 20, 23, 36]). Reproductive biology, sex cell structure and their behaviour have not yet been investigated in any member of the "basal” araphids, including those in Rhaphoneidaceae, Plagiogrammaceae and *Asterionellopsis*-like species. This clearly hampers our understanding of evolution of pennates because sexuality in "basal" araphid pennates, as opposed to their descendants, the "core" araphids and raphid diatoms, remains unknown.

The Family Plagiogrammaceae de Toni is a small diatom family, currently including approximately a dozen genera. Phylogenetic affiliation of the family to polar centrics and/or araphid pennates has been debated [27, 37], but recent molecular phylogenies firmly placed the family’s genera among pennates [32, 33, 35]. Some genera (*Dimeregramma* [J. Ralfs], *Plagiogramma* [R.K. Greville], *Glyphodesmis* [R.K. Greville]) have been placed in this family since the 1800s, with others added more recently. For example, several new or re-defined genera (*Talaroneis* [Kooistra & De Stefano], *Psammogramma* [S. Sato & Medlin], *Psammoneis* [S. Sato, Kooistra & Medlin], *Neofragilaria* [Desikachary, Prasad & Prema] and *Orizaformis* [Witkowski, Chunlian Li & Ashworth]) have recently been added to the family based on combined valve-morphological and molecular data [13, 32, 33]. To the best of our knowledge, sexual reproduction has not been reported in the published literature for any member of the Plagiogrammacean diatoms. If their phylogenetic position at the base of pennates, among the “basal” araphids, remains correct, then they are well positioned to provide information about characters ancestral to all pennates.

The goal of this paper is threefold. First, we describe the process of sexual reproduction in species from the family Plagiogrammaceae, one of the members of the “basal” araphids. Second, we compare their auxospore development and wall structure to that of other polar diatoms (centric and pennate). Finally, we discuss our findings in the context of the evolutionary history of these diatoms.

## Materials and methods

### Establishment and growth of stock cultures

Samples were collected from muddy-sand flats in the upper intertidal at various sites in New Brunswick and British Columbia, Canada, and the Caribbean island of Curaçao, the Netherlands (Table 1). Clonal chains were isolated to 12-well plates with f/20 growth media using the micropipette method [38], and kept at 12°C, under a light:dark cycle of 12:12 hours with mean ca. 33 μEm^-2^s^-1^ photons of light until growth was detectable. Once clones were established, they were transferred into a growth cabinet at 16–22°C, at the same light:dark cycle as before but with mean ca. 51 μEm^-2^s^-1^ photons of light, and gradually transferred to media with increasing nutrient concentration, up to f/2 [39]. Additionally, 9 clonal cultures (Table 1) used in a recent phylogenetic study of the family Plagiogrammaceae [32] provided by M. Ashworth and A. Witkowski were morphometrically examined and used in mating experiments when warranted by genetic similarity. Methods of collection, isolation and growth for these cultures are given in [32].

**Table 1:** Taxon name, collection information, and accession numbers for sequences and vouchers.

				Accession Numbers			
Taxon	Clone	Site*	Collection Date	18S	*rbc*L	ITS	BOLD
**Clones from author’s lab**							
*Delphineis* sp.	CCMP1095	na	na	KX586233	KX586257	KX586281	PLAGI024-16
*Dimeregramma acutumontgo*	Van5:3	3	19/05/2010	KX586237	KX586261	KX586285	PLAGI020-16
*D*. *acutumontgo*	StA:5	4	01/05/2010	KX586236	KX586260	KX586284	PLAGI021-16
*Dimeregramma* aff. *minus*	Dimere A1	5	22/05/2010	KX586235	KX586259	KX586283	PLAGI022-16
*Dimeregramma* aff. *minus*	Dimere A2	5	22/05/2010	KX586234	KX586258	KX586282	PLAGI023-16
*Plagiogramma staurophorum*	StA:2	4	01/05/2010	KX586255	KX586279	KX586302	PLAGI015-16
*P*. *staurophorum*	StA:3	4	01/05/2010	KX586254	KX586278	KX586301	PLAGI016-16
*P*. *staurophorum*	StA:6	4	01/05/2010	KX586253	KX586277	KX586300	PLAGI017-16
*P*. *staurophorum*	StA:7	4	01/05/2010	KX586252	KX586276	KX586299	PLAGI018-16
*P*. *staurophorum*	StA:8	4	01/05/2010	KX586256	KX586280	KX586303	PLAGI019-16
*P*. *tsawwassen*	Van3:10	1	30/04/2010	KX586238	KX586262	KX586286	PLAGI001-16
*P*. *tsawwassen*	Van4:1	2	30/04/2010	KX586243	KX586267	KX586291	PLAGI002-16
*P*. *tsawwassen*	Van4:3	2	30/04/2010	KX586244	KX586268	na	PLAGI014-16
*P*. *tsawwassen*	Van4:5	2	30/04/2010	KX586242	KX586266	KX586290	PLAGI003-16
*P*. *tsawwassen*	Van4:7	2	30/04/2010	KX586241	KX586265	KX586289	PLAGI004-16
*P*. *tsawwassen*	Van4:8	2	30/04/2010	KX586240	KX586264	KX586288	PLAGI005-16
*P*. *tsawwassen*	Van4:11	2	30/04/2010	KX586239	KX586263	KX586287	PLAGI006-16
*P*. *tsawwassen*	Van4:12	2	30/04/2010	KX586251	KX586275	KX586298	PLAGI007-16
*P*. *tsawwassen*	Van5:1	3	19/05/2010	KX586250	KX586274	KX586297	PLAGI008-16
*P*. *tsawwassen*	Van5:2	3	19/05/2010	KX586249	KX586273	KX586296	PLAGI009-16
*P*. *tsawwassen*	Van5:4	3	19/05/2010	KX586248	KX586272	KX586295	PLAGI010-16
*P*. *tsawwassen*	Van5:5	3	19/05/2010	KX586247	KX586271	KX586294	PLAGI011-16
*P*. *tsawwassen*	Van5:6	3	19/05/2010	KX586246	KX586270	KX586293	PLAGI012-16
*P*. *tsawwassen*	Van5:12	3	19/05/2010	KX586245	KX586269	KX586292	PLAGI013-16
**Clones from other studies**							
*Dimeregramma* sp.	HK288	6	03/2010	JN975244	JN975258	na	na
*Dimeregramma* sp.	HK358	7	06/2011	JX401231	JX401249	na	na
*Dimeregramma* sp.	HK359	8	10/2010	JX401232	JX401250	na	na
*Dimeregramma* sp.	HK376	9	06/2012	KF701596	KF701605	na	na
*Dimeregramma* sp.	SZCZCH915	10	15/10/2014	KT119332	KT119337	na	na
*Dimeregramma* sp.	SZCZP42	11	04/04/2013	KR048187	KR048208	na	na
*Dimeregramma* sp.	SZCZP43	12	17/12/2012	KR048186	KR048209	na	na
*Dimeregramma* sp.	SZCZP256	13	10/04/2013	KR048190	KR048210	na	na
*Dimeregramma* sp.	SZCZP475	11	04/04/2013	KR048189	KR048207	na	na
*Plagiogramma* aff. *staurophorum*	HK212	14	06/2008	HQ912656	HQ912520	na	na
*Plagiogramma* sp.	HK324	15	07/2011	JX413546	JX413563	na	na
*Plagiogramma* sp.	HK374	9	na	KF701594	KF701603	na	na
*Plagiogramma* sp.	HK410	9	na	KJ577867	KJ577904	na	na
*Plagiogramma* sp.	SZCZCH437	10	15/10/2014	KR048188	KR048206	na	na

*Sites: 1 = Ucluelet, Vancouver Island, BC (48.9482° N 125.5534° W); 2 = Tofino, Vancouver Island, BC (49.1549° N 125.9055° W); 3 = Tsawwassen, BC (49.0228° N 123.1056°W); 4 = Indian Point, St. Andrews, NB (45.0689° N 67.0411°W); 5 = Curaçao, Netherlands (12.1044°N 68.9412°W); 6 = Port Aransas, TX; 7 = Baffin Bay, TX; 8 = St. George Island, FL; 9 = Hunting Island, SC; 10 = Korea (34.93° N 128.42° E); 11 = South Africa (33.15° S 18.03° E); 12 = La Gomera Beach, Canary Islands, Spain; 13 = Namibia (22.98° S 14.47° E); 14 = Taelayag Beach, Guam, USA; 15 = Potlatch State Park, WA; na = not available

### Sexual induction protocol

Exponentially growing cultures (15–16°C, 12:12 light:dark, mean ca. 51 μEm^-2^s^-1^ photons of light) were sexualised by placing a pair of clones in a well of a sterile 12-well plate filled with 3 mL of the f/10 or f/2 medium and placed in a growth chamber at 12°C under a light:dark cycle of 12:12 hours with a mean of ca. 33 μEm^-2^s^-1^ photons of light. On the second day at 12°C, the mating plate was shaded to a mean irradiance of ca. 11 μEm^-2^s^-1^ under the same thermal and light cycle regime. For each mating pair, two wells were designated as a control, each holding only one of the clones. Sexual identity of individual clones in specific interactions was determined by pair-wise crossing in 2011–2012, 2013 and 2016. Each pair was mated at least three times.

### Light microscopy for images and videos

Brightfield and epifluorescence light microscopy were performed using Zeiss microscopes (Carl Zeiss, Oberkochen, Germany) as required. Sex cells were time-lapse recorded live using a Zeiss Axiovert 200 inverted microscope equipped with a QImaging Micropublisher 3.3 RTV camera and QCapture Pro software (QImaging, Burnaby, BC).

To visualize nuclei in sexualised cells and in auxospores, cells were fixed with 2.5% (v/v) glutaraldehyde in seawater (final concentration) and stained with DAPI (4’,6-diamidino-2-phenylindole) according to [40] with minor modifications. Chloroplasts were bleached with 10 mL 99% methanol followed by 5 mL Tris buffer. Following bleaching, 0.1 μl DAPI (10 μg/mL) was added and incubated in darkness at 2–3°C for another 24 hours prior to examination. Alternatively, as required and in combination with PDMPO staining described below, wet mounted live cells were treated with 1 drop of Vectashield antifade mounting medium with DAPI (Vector Laboratories, Burlingame, CA) before coverslipping, and allowed to incubate for 10–15 minutes before examination.

Wall silicification was visualized by tracing incorporation of PDMPO (2-(4-pyridyl)-5-((4-(2-dimethylaminoethylaminocarbamoyl)methoxy)phenyl)oxazole), Thermo Fisher Scientific, Waltham, MA) into developing vegetative cell walls and auxospores. A stock solution of 100 μM PDMPO in distilled water was added to the mating culture to a final dilution of 0.125 μM at the time the parent clones were mixed, to allow its incorporation into developing siliceous constituents of their cell walls [41] or following mixing of the parents if the initial cell walls were of interest. Sexual identity of the very small clones (in 2016) was examined by growing one of the parents with PDMPO for one day prior to combining them in a mating well. The PDMPO-treated clone was therefore marked by the fluorophore in the newly deposited valves, then carefully washed immediately prior to adding it to the well with the compatible partner, and subjected to the sexual induction protocol. The association of paired auxospores could thus determine clone identity with their parental chain containing PDMPO marked valves. PDMPO protocol is available at dx.doi.org/10.17504/protocols.io.huub6ww.

Brightfield and epifluorescence light microscopy were performed using two Zeiss microscopes as required. A Zeiss Axioskop 2 Plus fitted with a cooled AxioCam color camera, HBO 100 fluorescence illuminator and Filter Set 01 was used for reconnaissance work, while a Zeiss AxioImager.Z2 microscope with a cooled AxioCam MRm monochrome camera, Colibri LED fluorescence illuminator (365 nm LED) and Filter Sets 62HE and 49 was used for in-depth investigation. Monochrome fluorescence images presented here were pseudo-colored appropriately based on the filter used for acquisition.

### Scanning electron microscopy (SEM) and energy dispersive x-ray spectroscopy (EDS)

Frustules from each of the clonal cultures were prepared for SEM examination following [42], within a few months after culture establishment. Lightly and non-silicified cells (gametes and young auxospores) were fixed with 2.5% glutaraldehyde in f/2 media, rinsed 4X with f/2 media (~100 mL) every 10 min, with gentle vacuum filtering between solution changes onto a 3 μm pore size polycarbonate filter (Sterlitech Corporation, Kent, WA) in a filtration tower. Specimens were then partially dehydrated following the same protocol as above using 20%, 50%, and 70% ethanol:distilled water. Filters were freeze-dried overnight at ca. 7 Pa with phosphorus pentoxide in the freeze drier chamber as an additional drying agent. The dried filters were mounted on aluminum stubs with double-sided tape, rimmed with colloidal carbon, and coated with a ca. 15 nm layer of gold using a Hummer 6.2 sputtering unit (Anatech Ltd., Union City, CA). Images were acquired using a JEOL JSM-5600 scanning electron microscope (JEOL USA, Peabody MA), at a working distance of 8–10 mm and 10 kV accelerating voltage.

EDS was performed with the same instrument equipped with an Oxford Inca Energy 200 EDS system (Oxford Instruments, High Wycombe, UK) at 20 mm working distance. Since the only element of interest in this study was silicon (Si-K_α_, X-ray energy 1.74 keV), an accelerating voltage of 10 kV provided sufficient overvoltage for efficient X-ray excitation. Spectra were acquired for 100 s (dead time corrected) at 0.1 nA beam current, energy range 0–10 keV into 1024 channels. The EDS spectra were collected from intact and unobstructed structures and/or auxospores. Spectra from the polycarbonate support filter adjacent to the auxospores were also routinely taken and showed no remote excitation from neighbouring siliceous components (if present) at distances as close as 3 μm.

Standard quantifiable characters used in diatom morpho-taxonomy were collected for a minimum of 12 valves for each of our clones using dmfMeasure software ([43]; Table 2). For the five most sexually active clones, metrics were also recorded for diminished valves grown in culture for approximately 6 years and showing various culture induced abnormalities (indicated as “small” in Table 2). These measurements are excluded from species descriptions (as based on old cultures) but included in Table 2 and relevant figures for comparison. Valve structure terminology follows [27], while terminology associated with reproduction follows [44].

**Table 2:** Clone morphometrics in 2010–11, when our cultures were established. Valves of the best mating pairs were also measured approximately six years after clones were established (denoted as “small”), and their progeny indicated by parental clone codes separated by “x”. Values presented as: mean (standard deviation) [N]. GenBank clones were measured in part by the authors of [32] and by us in 2016.

Taxon	Clone	Valve Length (μm)	Valve Width (μm)	Striae (in 10 μm)	Pores (in 10 μm)
**Clones from author’s lab**					
*Dimeregramma acutumontgo*	Van5:3	24.9 (0.99) [12]	5.7 (0.23) [12]	13.8 (0.53) [12]	16.4 (1.61) [12]
*D*. *acutumontgo*	StA:5	29.6 (2.04) [12]	8.5 (0.41) [12]	12.3 (0.45) [12]	15.3 (1.25) [12]
*D*. *acutumontgo*	StA:5 small	5.8 (1.09) [18]	3.8 (0.65) [14]	14.9 (2.38) [17]	19.0 (3.47) [15]
*D*. *acutumontgo*	Van5:3xStA:5	66.0 (9.45) [4]	6.7 (1.00) [4]	12.9 (na) [1]	14.2 (na) [1]
*Dimeregramma* aff. *minus*	Dimere A1	6.7 (0.57) [22]	4.3 (0.36) [19]	19.7 (1.48) [23]	21.9 (2.06) [23]
*Dimeregramma* aff. *minus*	Dimere A2	6.7 (0.57) [19]	4.4 (0.27) [16]	20.4 (1.53) [17]	21.3 (1.12) [18]
*Plagiogramma staurophorum*	StA:2	35.8 (0.85) [17]	7.6 (0.22) [18]	11.2 (0.36) [18]	11.3 (0.58) [18]
*P*. *staurophorum*	StA:3	22.7 (0.48) [19]	6.6 (0.34) [18]	11.3 (0.41) [20]	11.2 (0.49) [20]
*P*. *staurophorum*	StA:6	48.7 (1.15) [17]	9.3 (0.43) [16]	9.2 (1.14) [18]	10.2 (1.15) [18]
*P*. *staurophorum*	StA:7	20.7 (1.03) [5]	6.5 (0.44) [6]	11.3 (0.38) [8]	11.2 (0.45) [8]
*P*. *staurophorum*	StA:8	16.4 (0.71) [19]	6.5 (0.41) [17]	11.6 (0.62) [18]	11.2 (0.55) [18]
*P*. *staurophorum*	StA:7 small	9.2 (0.60) [12]	6.8 (0.57) [12]	10.0 (0.79) [12]	14.1 (1.98) [12]
*P*. *staurophorum*	StA:8 small	8.0 (0.82) [12]	6.6 (0.83) [12]	10.4 (0.87) [12]	14.7 (1.62) [12]
*P*. *staurophorum*	StA:7x8	66.5 (4.93) [10]	7.0 (0.69) [10]	10.0 (1.04) [10]	12.1 (1.66) [10]
*P*. *tsawwassen*	Van3:10	22.1 (0.92) [17]	5.8 (0.23) [17]	15.0 (0.56) [18]	14.9 (0.67) [18]
*P*. *tsawwassen*	Van4:1	10.1 (0.38) [19]	5.1 (0.22) [15]	16.3 (0.92) [16]	15.7 (0.90) [16]
*P*. *tsawwassen*	Van4:3	27.4 (0.60) [16]	5.9 (0.25) [16]	15.2 (0.53) [16]	15.3 (0.66) [16]
*P*. *tsawwassen*	Van4:5	18.9 (0.86) [16]	5.2 (0.26) [16]	15.4 (0.39) [16]	15.1 (0.54) [16]
*P*. *tsawwassen*	Van4:7	11.6 (0.70) [19]	5.9 (0.51) [17]	16.0 (0.76) [18]	15.7 (1.05) [18]
*P*. *tsawwassen*	Van4:8	12.2 (0.79) [19]	5.9 (0.57) [16]	16.4 (0.92) [17]	15.4 (0.68) [17]
*P*. *tsawwassen*	Van4:11	13.4 (0.88) [19]	5.7 (0.35) [16]	16.0 (0.93) [16]	14.9 (0.45) [16]
*P*. *tsawwassen*	Van4:12	10.4 (0.94) [18]	5.2 (0.39) [16]	16.1 (0.99) [16]	15.4 (0.66) [16]
*P*. *tsawwassen*	Van5:1	29.2 (1.01) [17]	6.2 (0.42) [16]	15.2 (0.44) [15]	15.5 (0.49) [15]
*P*. *tsawwassen*	Van5:2	29.6 (0.59) [14]	6.1 (0.33) [14]	15.1 (0.46) [15]	15.4 (0.69) [15]
*P*. *tsawwassen*	Van5:4	29.3 (0.58) [13]	6.3 (0.38) [13]	14.7 (0.54) [13]	15.5 (0.54) [13]
*P*. *tsawwassen*	Van5:5	28.6 (1.10) [12]	6.1 (0.22) [12]	13.0 (0.17) [12]	16.0 (1.05) [12]
*P*. *tsawwassen*	Van5:6	18.0 (1.01) [12]	5.9 (0.26) [12]	13.8 (0.61) [12]	15.5 (2.25) [12]
*P*. *tsawwassen*	Van5:5 small	6.7 (1.16) [21]	4.5 (0.54) [16]	14.1 (1.34) [21]	18.5 (2.50) [21]
*P*. *tsawwassen*	Van5:6 small	5.3 (0.64) [20]	4.3 (0.36) [16]	14.9 (1.88) [21]	18.8 (3.87) [18]
*P*. *tsawwassen*	Van5:5x5:6	76.4 (6.51) [12]	4.7 (0.62) [12]	13.2 (0.23) [12]	19.0 (2.77) [12]
*P*. *tsawwassen*	Van5:12	19.2 (0.77) [16]	6.1 (0.60) [16]	15.6 (0.48) [19]	15.2 (0.65) [19]
**GenBank clones**					
*Dimeregramma* sp.	HK288	21.7 (1.55) [6]	6.0 (0.31) [7]	14.4 (0.71) [7]	18.9 (3.55) [7]
*Dimeregramma* sp.	HK358	12.9 (3.62) [9]	6.7 (0.75) [9]	10.1 (0.50) [6]	11.5 (0.58) [4]
*Dimeregramma* sp.	HK359	13.5 (3.59) [14]	5.2 (0.87) [14]	10.8 (0.56) [14]	15.0 (1.96) [13]
*Dimeregramma* sp.	HK376	12.2 (3.58) [5]	5.5 (0.65) [5]	11.0 (0.25) [4]	14.2 (1.26) [3]
*Dimeregramma* sp.	SZCZCH915	14.5 (6.36) [19]	5.7 (0.61) [18]	13.9 (0.61) [19]	17.0 (1.61) [18]
*Dimeregramma* sp.	SZCZP256	10.4 (2.54) [12]	6.6 (0.66) [12]	13.6 (0.63) [12]	17.0 (2.23) [12]
*Dimeregramma* sp.	SZCZP475	8.2 (2.95) [24]	4.7 (0.92) [21]	14.3 (1.30) [24]	19.6 (3.97) [22]
*Plagiogramma* sp.	HK324	21.1 (3.57) [10]	7.1 (0.40) [8]	13.7 (0.30) [10]	16.2 (1.70) [10]
*Plagiogramma* aff. *staurophorum*	HK212	38.4 (1.75) [13]	18.3 (2.33) [11]	7.7 (1.05) [13]	7.8 (0.91) [13]

na = not available

### Molecular analyses

Cells were harvested in the exponential growth phase and their DNA extracted using an UltraClean Soil DNA Kit (MoBio Laboratories, Carlsbad, CA). DNA was obtained from 23 monoclonal cultures established for this study. DNA sequences of 14 strains used in [32] and available in GenBank were also included into our analyses. Three DNA fragments were amplified with specific primers for a ~400 bp conservative region of the nuclear encoded 18S rDNA gene including the V3 and part of the V2 variable regions and ending just before the V4 region (18S; [45]); a ~800 bp fragment of the internal transcribed spacer region (ITS; [46]); and a ~900 bp fragment of the gene coding for the large subunit of ribulose-1,5-bisphosphate carboxylase oxygenase (*rbc*L) of the chloroplast genome [46]. Each 25 μL PCR reaction mixture, containing 12.5 μL of GoTaq Green (Promega, Madison, WI), 1.25 μL each of the forward and reverse primers, and 10 μL of DEPC water (Invitrogen, Carlsbad, CA), was subjected to 35 rounds of thermal cycling as described by [45] for 18S, and [46] for ITS and *rbc*L. PCR fragments were purified and sequenced at McGill University and Genome Québec Innovation Centre, Montréal. Sequences were edited and aligned using BioEdit [47] with optimal alignment parameters based on those of [48–50]. Sequences were cleaned and trimmed to the following aligned lengths: 444 bp for the 18S fragment, 881 bp for the ITS region, and 749 bp for *rbc*L. Alignments for phylogenetic analysis were performed using the program MUSCLE [51]. Maximum Likelihood (ML) trees were constructed using Randomized Accelerated Maximum Likelihood (RAxML v. 8.2.0; [52]) with a general time-reversible model of nucleotide substitution using four discrete rate categories to approximate a gamma distribution, and 1000 bootstrap replicates. Multi-gene trees were made by concatenating alignments of the three genes using SequenceMatrix [53], with leading and trailing gaps represented as undefined characters. Outgroups for all markers were the strain *Delphineis* sp. CCMP1095, with the addition of GenBank sequences for *Asterionellopsis* cf. *glacialis* CCMP139 and *Asteroplanus* aff. *karianus* (CCMP1717; [9]). Tree topologies were also validated by Bayesian analysis using MrBayes v.3.2.6 [54], using the same nucleotide substitution model as described for RAxML. Bayesian posterior probabilities were computed by running four chains for 1,000,000 generations using the program default priors. Trees were sampled every 100 generations in two independent runs. Two thousand five hundred trees were discarded from the burn-in phase of the analysis, so that only trees in the stationary phase of the run were considered. The main purpose of the phylogenetic analyses was to confirm the taxonomic identity of the clones examined.

### Nomenclature

The electronic version of this article in Portable Document Format (PDF) in a work with an ISSN or ISBN will represent a published work according to the International Code of Nomenclature for algae, fungi, and plants, and hence the new names contained in the electronic publication of a PLOS ONE article are effectively published under that Code from the electronic edition alone, so there is no longer any need to provide printed copies.

The online version of this work is archived and available from the following digital repositories: PubMed Central, LOCKSS.

## Results

Among 23 clones established for this study, 21 contributed to three syngens corresponding to three species: two *Plagiogramma* (*P*. *staurophorum* and *P*. *tsawwassen*) and one *Dimeregramma*, *D*. *acutumontgo*. Two clones did not sexualise. Taxonomic discussion of the syngens’ identity and their biological, molecular and morphological characteristics are presented further below.

### Observations of sexualised live cells (Figs 1 and 2)

**Fig 1:**
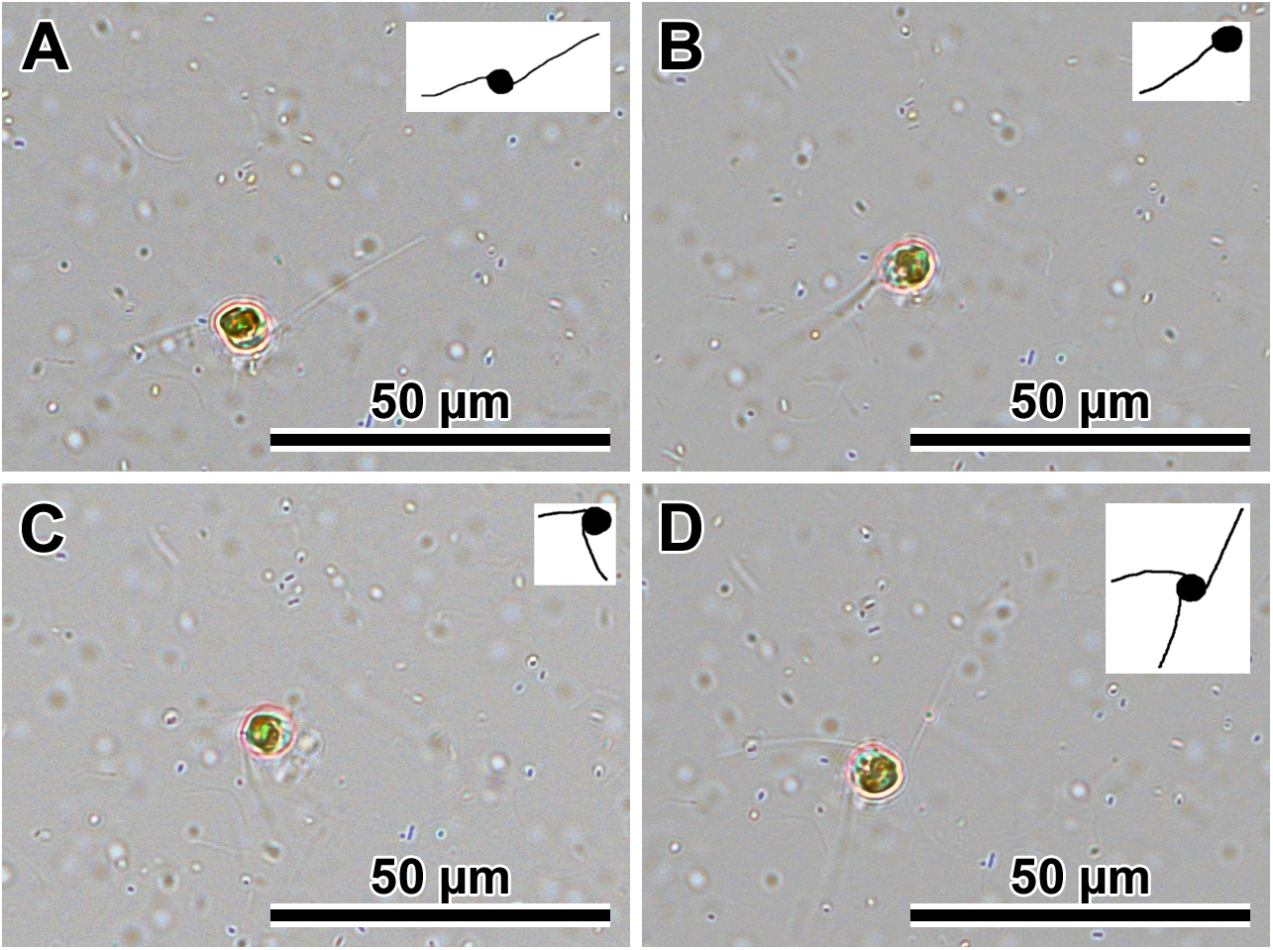
Free males from *P*. *tsawwassen* Van4:1 male clone showing extruded and retracted multiple pseudopodia. (A through D) The same cell was captured as 32 individual frames over 16 minutes (only selected frames are presented). Inset for each image shows outlines of cell and pseudopodia for clarity.

**Fig 2:**
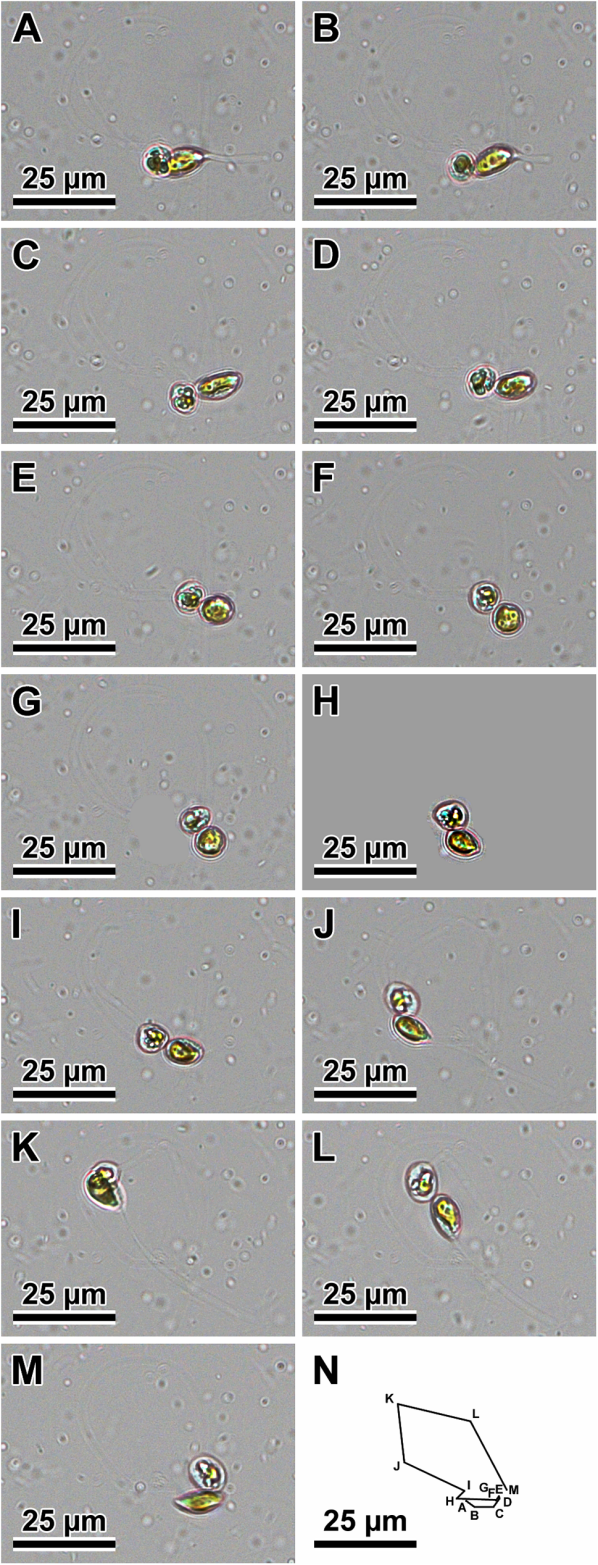
An example of two free, vigorously motile males of *P*. *tsawwassen* clone Van4:1. (A through M) Note change in cell shape and extruded pseudopods. (N) Cumulative summary of the track of the male; letters associated with points in the track correspond to images A through M.

The mode of sexual reproduction observed in the clones of two *Plagiogramma* species and in *Dimeregramma acutumontgo* examined here was identical in all stages of the process. Gametangia were distinguishable from vegetative cells by their greater pervalvar axis relative to cells before mitosis. Gametangia dehisced to liberate two secondary meiocytes. In all our species, these cells (products of Meiosis I) were motile. The vigour of motility of these cells differed greatly, depending on sex and clone. Some clones produced cells that vacated the gametangia as a pair or individually and were vigorously motile, moving freely and randomly about the environment. We designated them as males. Motility in these cells was associated with extrusion and retraction of pseudopodia; live cells with one to three pseudopodia are illustrated (Fig 1). While in motion, the cell shape changed from spherical to elongated ellipsoid (Fig 2). An example of a vigorous male movement path is shown in Fig 2N. The other sex produced far less vigorously motile cells. Cells of this second type were most vigorous when vacating the gametangium, sluggishly shuffling about thereafter while staying together, perched on the opening of one of the parental thecae. We observed no pseudopodia on these cells. We designated these as females.

Following syngamy (observed on several occasions), the resulting pair of zygotes remained attached to the maternal theca. Then, tubular auxospores developed with no evidence of incunabula in LM images. Single auxospores were infrequent, generally associated with an aborted gamete and/or auxospore. Chloroplasts were appressed to the auxospore walls and dispersed throughout, except for the apices. The longest auxospores were straight or crescent-shaped. The presence of the initial frustule within the auxospore was notable by its rigid appearance. It is unclear how the initial cell vacated the auxospore wall, but the walls ought to have been rather flimsy when the initial frustule was completed because they appeared thin and transparent.

### Nuclear behaviour during gametogenesis and auxospore development (Figs 3 and 4)

**Fig 3:**
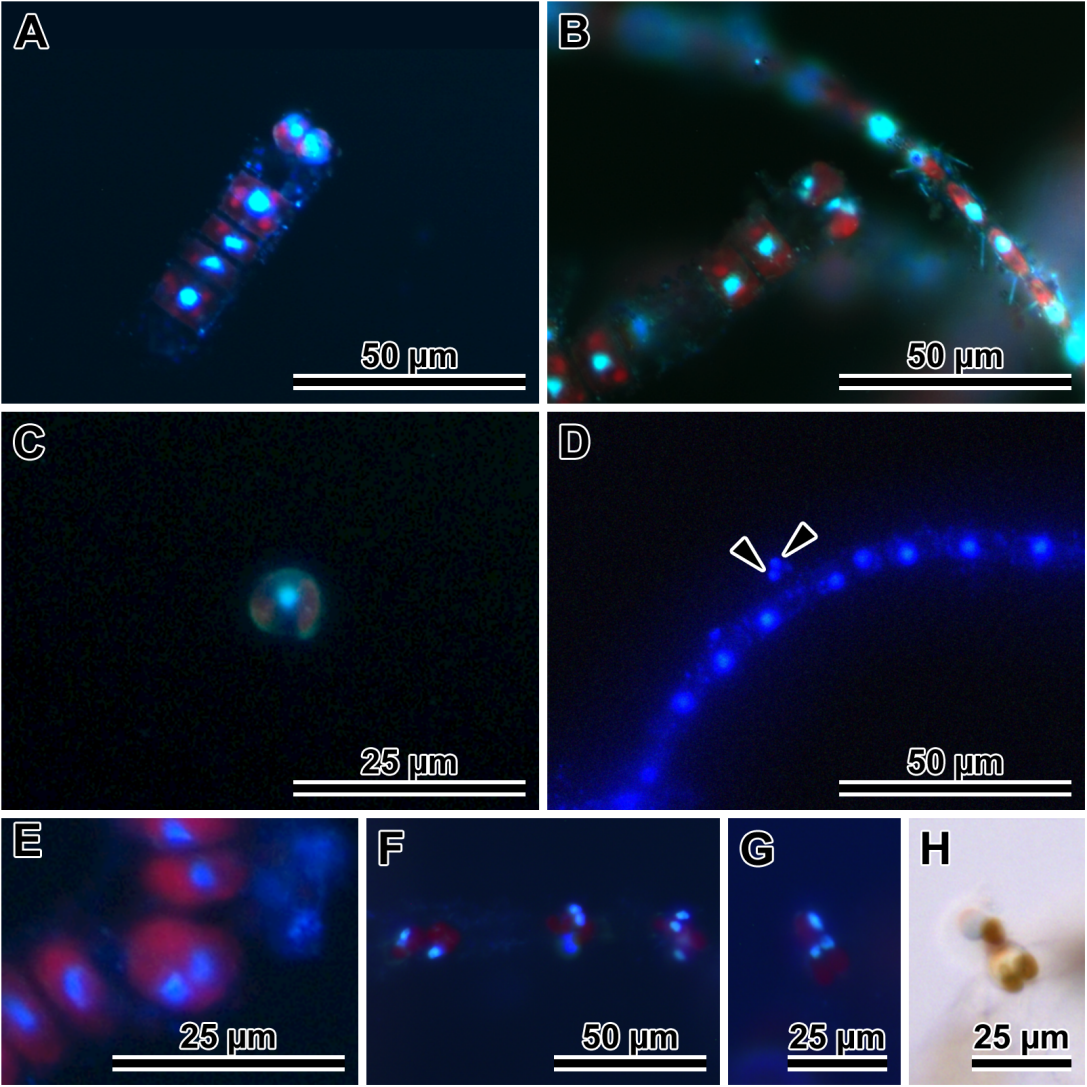
Gamete development and behaviour in *P*. *tsawwassen*. **(**A and B) Stages in female gamete development. (A) A pair of uni-nucleate secondary oocytes. (B) Bi-nucleate cells destined female gametes, both characteristically perched on maternal valve copula. (C and D) Stages in male gamete development. (C) A uni-nucleate secondary spermatocyte liberated from paternal theca. (D) Bi-nucleate (arrowheads) male gamete free in mating dish environment. (E) Primary meiocyte after Meiosis I and before cytokinesis. (F) Three female gametangia with mostly bi-nucleate gametes perched on the thecae. (G) Gamete fusion, note bi-nucleated female gamete and uni-nucleate secondary spermatocyte in DAPI. (H) The same pair of live cells in LM; DAPI stained nuclei are blue, red is natural autofluorescence of chloroplasts.

**Fig 4:**
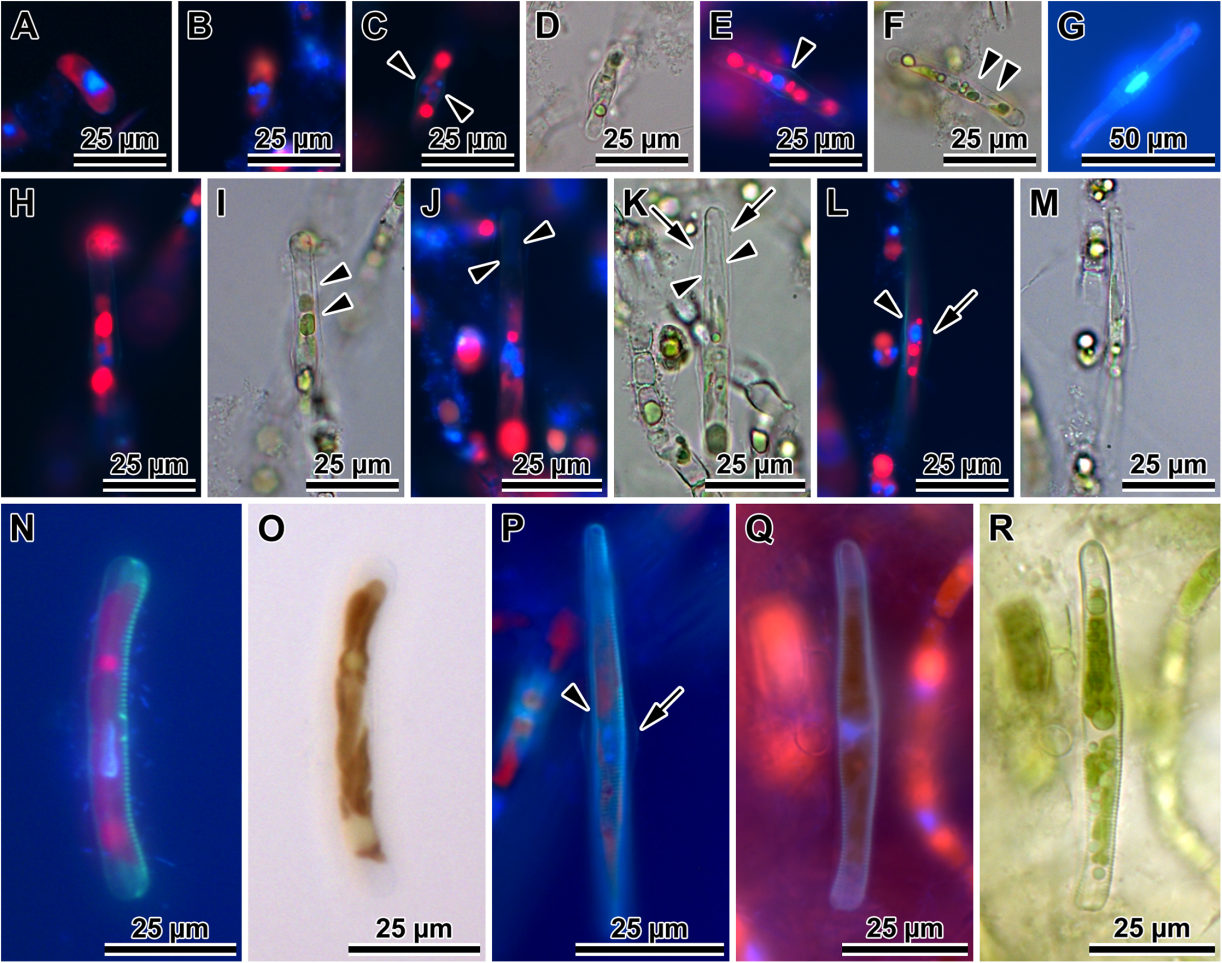
Nuclear behavior and PDMPO-traced silicification of auxospore wall and initial valves in *P*. *tsawwassen*. (A) Multi-nucleated, small, ellipsoidal auxospore, 3 nuclei visible in the focal plane. (B) Another multi-nucleated, small ellipsoidal auxospore with 3 or 4 nuclei, silica cannot yet be visualized in this auxospore wall. (C) PDMPO-stained, older auxospore with attenuated ends and 3 or possibly 4 nuclei and primary band of transverse perizonium clearly visible (arrowheads) as thin, greenish lines bracketing auxospore mid section. (D) Corresponding brightfield image of C. (E) PDMPO-stained perizonium (arrowhead) in a larger, more tubular auxospore with a single nucleus. (F) Corresponding brightfield image of E, transverse perizonial bands visible as slight indentations (between arrowheads). (G) Clearly visible transverse perizonial bands. PDMPO in this image appears light blue due to strong concentration of Vectashield/DAPI. (H) A full size, PDMPO-stained, bi-nucleate auxospore, focusing on silicified transverse perizonium. (I) Corresponding brightfield image of H, also with well resolved bands of transverse perizonium (arrowheads). (J) Another bi-nucleated, full size auxospore with PDMPO-stained transverse perizonium and initial epivalve (arrowheads point to valve edges). (K) Corresponding brightfield image of J, note distant position of the transverse perizonium (arrows). Arrowheads point to valve edges. (L) A full size auxospore, slightly turned sideways with lightly silicified initial epivalve (strongest green strip; arrowhead) and transverse perizonium (arrow). (M) Corresponding brightfield image of L. (N) A mature auxospore with initial frustule showing lightly silicified PDMPO-stained initial epivalve and strongly silicified initial hypovalve. (O) Corresponding brightfield image of N. (P) A mature auxospore with initial frustule in cingular orientation showing different degree of silicification of the transverse perizonium (arrow), initial epivalve (arrowhead) and initial hypovalve with distinct striation. (Q) A post-sexual cell with more typical frustule, both valves strongly silicified and showing strong striation. (R) Corresponding brighfield image of Q.

DAPI staining gave information about the behaviour of nuclei in gametangia, gametes (Fig 3) and in developing auxospores (Fig 4). Gametangia grew and then underwent Meiosis I (Fig 3E). This division was followed by cytokinesis (Fig 3A, 3C and 3F). In the majority of cells, two secondary meiocytes underwent acytokinetic Meiosis II after leaving their gametangium (Fig 3B, 3D and 3F). The many cells found free in the environment (thought to be male sex cells; Figs 1, 2A, 3C, 3G and 3H) were still uni-nucleate. Hereafter, only cells containing two nuclei will be considered gametes, as these are the ones with the capacity to deliver a gametic nucleus to a receptive female nucleus, with the supernumerary nucleus eventually becoming pyknotic. However, even uni-nucleated secondary spermatocytes were observed to fuse with bi-nucleated female gametes, leading to tri-nucleate cells with unequal-size nuclei; Fig 3G and 3H show a large nucleus in the motile, free secondary meiocyte of the male gametangium and two small haploid nuclei in a female gamete perched on the maternal theca during their fusion. Cells from female gametangia were normally perched near the rim of an open theca (Fig 3B, 3F and 3G), and could be uni-nucleate (secondary meiocytes), or bi-nucleate (female gametes). It is unclear when exactly gametic nuclei fused and supernumerary nuclei pyknotized because bi-nucleated cells (both freely motile and "perched") most often contained nuclei of about equal size (Fig 3B, 3D, 3F and 3G). Multi-nucleated expanding auxospores were also often found (three and four small nuclei of about equal size can be seen in a common focal plane of this auxospore; Fig 4A, 4B and 4C). Quadri-nucleate auxospores are expected to occur in cases when pyknosis of the supernumerary nuclei is delayed and both male and female gametic sister nuclei co-exist in the same auxospore prior to functional gametic nuclei fusion. Quadri-nucleate auxospores were seen even after their expansion had begun (Fig 4C and 4D).

A single, large diploid nucleus was seen in medium and maximum size auxospores (Fig 4E and 4G). Bi-nucleate auxospores appeared again among the largest of them (Fig 4E, 4F, 4L, 4M, 4Q and 4R). Using PDMPO as a tracer of silica deposition in the auxospore wall (Fig 4C, 4E, 4H, 4J, 4L, 4N, 4P and 4Q), lightly silicified transverse perizonial bands were detected in auxospores of all sizes (Fig 4C, 4D, 4E, 4F and 4G). Following acytokinetic mitosis, an initial epivalve developed (Fig 4H, 4I, 4J and 4K), the supernumerary nucleus pyknotized, and the auxospore became uni-nucleate again (Fig 4L and 4M). In PDMPO-treated auxospores, the initial epivalve appeared as a thin, structureless strip of pale yellow (or green in preparations without the blue background from Vectashield) when the initial frustule could be seen in girdle orientation (Fig 4L, 4M, 4N and 4O). The initial hypovalve was more typical, and had a thicker basal silica-layer and visible striae. Striae and internal costae were clearly detectable when the initial hypovalve was in an orientation favourable for imaging (Fig 4P and 4R). The functional initial frustule contained a second normally heavily silicified valve, which became a new hypovalve (Fig 4Q and 4R). Supernumerary nuclei pyknotized following the deposition of each of the valves deposited within an auxospore.

### Auxospore wall fine structure (Figs 5–8)

**Fig 5:**
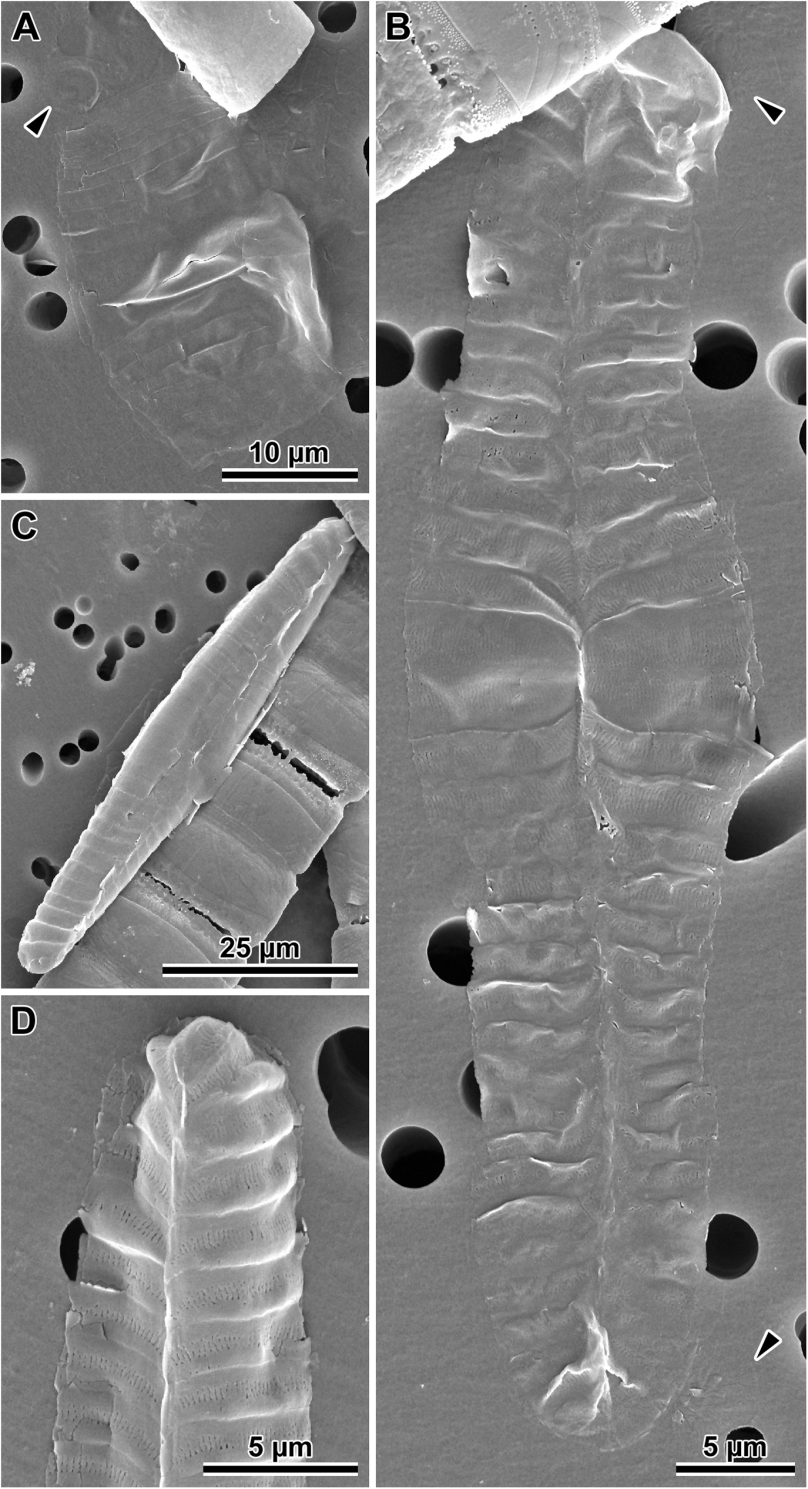
Auxospore developmental stages. (A) A set of transverse perizonial bands from a very young, ellipsoidal auxospore of *P*. *staurophorum*, delicate incunabular scales indicated at the ends of the distal perizonial bands (arrowhead). (B). Montage of five images of an older, tubular auxospore of *P*. *tsawwassen* in ventral orientation, perizonial band ends precisely abut each other, forming a notable seam or venter, note incunabular scale near apices (arrowheads). (C) Mature auxospore of *P*. *staurophorum* in a ventral orientation filled up by an initial frustule, showing subapical perizonial bands slanted towards the venter. (D) Apex of another mature auxospore of *P*. *tsawwassen* detailing pores and slits on the perizonial bands.

In SEM observations, auxospore developmental stages followed the general pattern seen in the majority of pennate diatoms examined. Initially zygotes were spherical, but as soon as they started expanding, they took on an ellipsoidal-progressing-to-tubular shape. There were no obvious incunabular apical caps, but very delicate incunabular scales could be seen in expanding auxospores (visible in *P*. *staurophorum* and *P*. *tsawwassen* SEM images, Fig 5A and 5B; arrowheads). Transverse perizonia expanded bi-directionally as the auxospore grew, seemingly by pushing out individual bands at the opposite apices. Multiple transverse bands in at least one of our species (*P*. *staurophorum*) were produced either all together, or at least in close succession one after and beneath another. Individual bands were organised in a manner similar to sections of a collapsible cup (Fig 5A), not added one at a time during auxospore expansion. Transverse perizonial bands were open (Fig 5B, 5C and 5D). The primary band was the widest and symmetrically structured, with two equally developed submarginal bands of short rows or irregular pores and slits, one on each side of the band median (Fig 5B). The ends of this band were gently rounded and abated but did not overlap. The consecutive bands (secondary, tertiary, etc.) were made of three parallel sections, two marginal (nearly structureless borders), and a central strip ornamented with irregularly punctuated slits similar to those present on the primary bands. The ends of these bands were slightly curved centripetally (abapically, or away from the apex), wrapping around the underlying neighbour. Transverse band ends lined up on the ventral side of the auxospore (Fig 5B and 5C) and neatly abated. Some of the transverse bands were visibly slanted, particularly near apices (Fig 5C). The auxospore apex was covered by a short band with structure similar to secondary bands (Fig 5D). Longitudinal perizonia structured as in [2] (figs. 4b, c and 5a) were not found.

**Fig 6:**
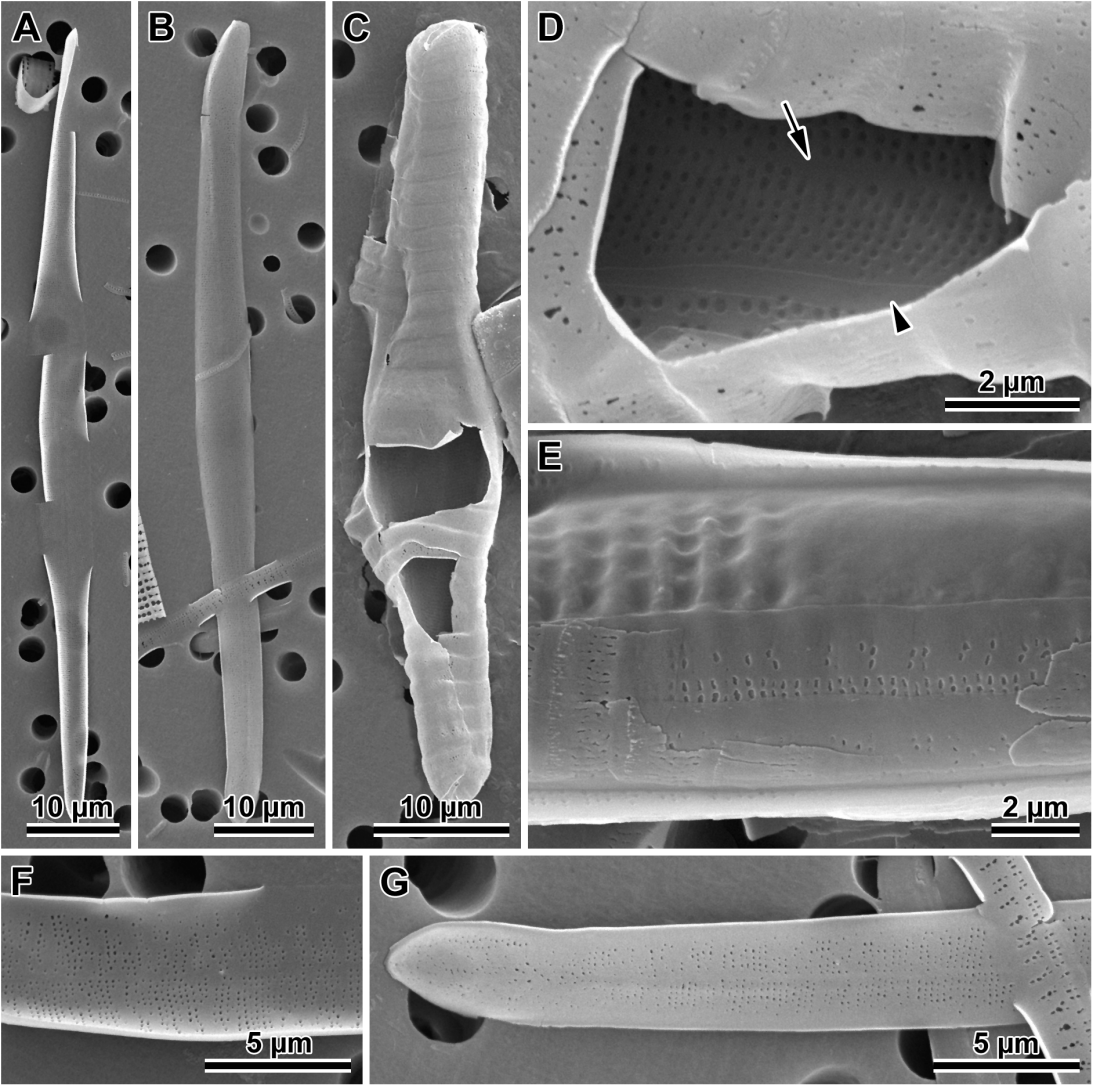
Initial valves. (A) Internal and (B) external view of an initial epivalve of *P*. *tsawwassen*. (C) Partially damaged mature auxospore of *P*. *staurophorum* exposing the initial epivalve. (D) Enlarged section of the auxospore from C, showing initial epivalve (arrow) and one girdle band (arrowhead). (E) Another auxospore of *P*. *staurophorum* with initial hypovalve deposited internally to initial epitheca, two initial epicopulae cradle this hypovalve. (F) Close up of the internal surface of an initial epivalve from A, illustrating simple pores in mid-section. (G) External view of the perforation type near initial epivalve apex and on a copula of *P*. *tsawwassen*.

**Fig 7:**
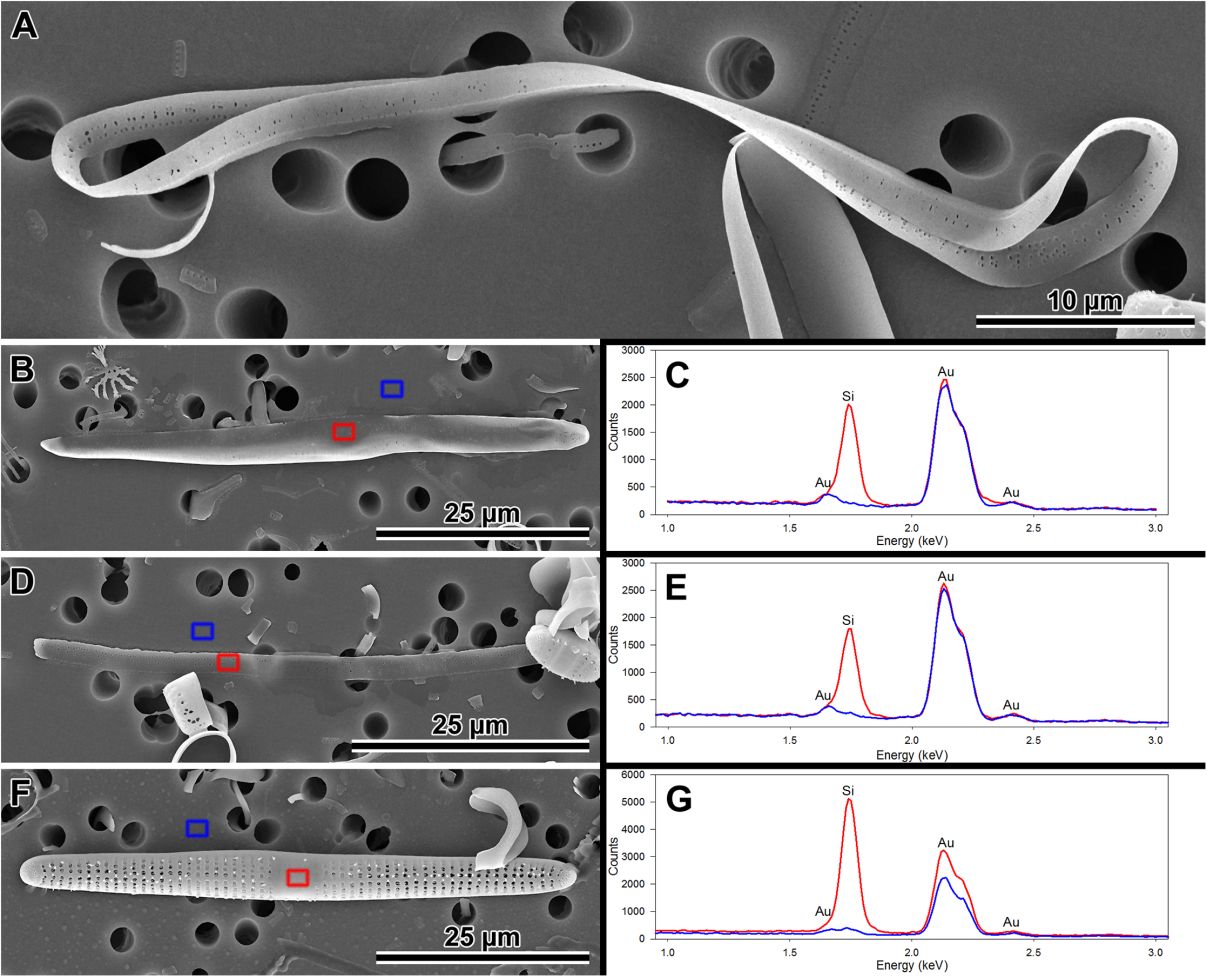
Morphology and silicification of initial frustule components of *P*. *staurophorum*. (A) Closed hoop of the initial band. (B) Initial epivalve. (C) Corresponding spectrum from B showing relatively weak degree of silicification. (D) Fragment of initial girdle band. (E) Corresponding spectrum from D showing degree of silicification similar to the initial valve in B and C. (F) A more typical, strongly silicified hypovalve. (G) Corresponding spectrum from F with silica peak nearly three times the height (note difference in y-axis scale) of those from initial epitheca in B through E.

**Fig 8:**
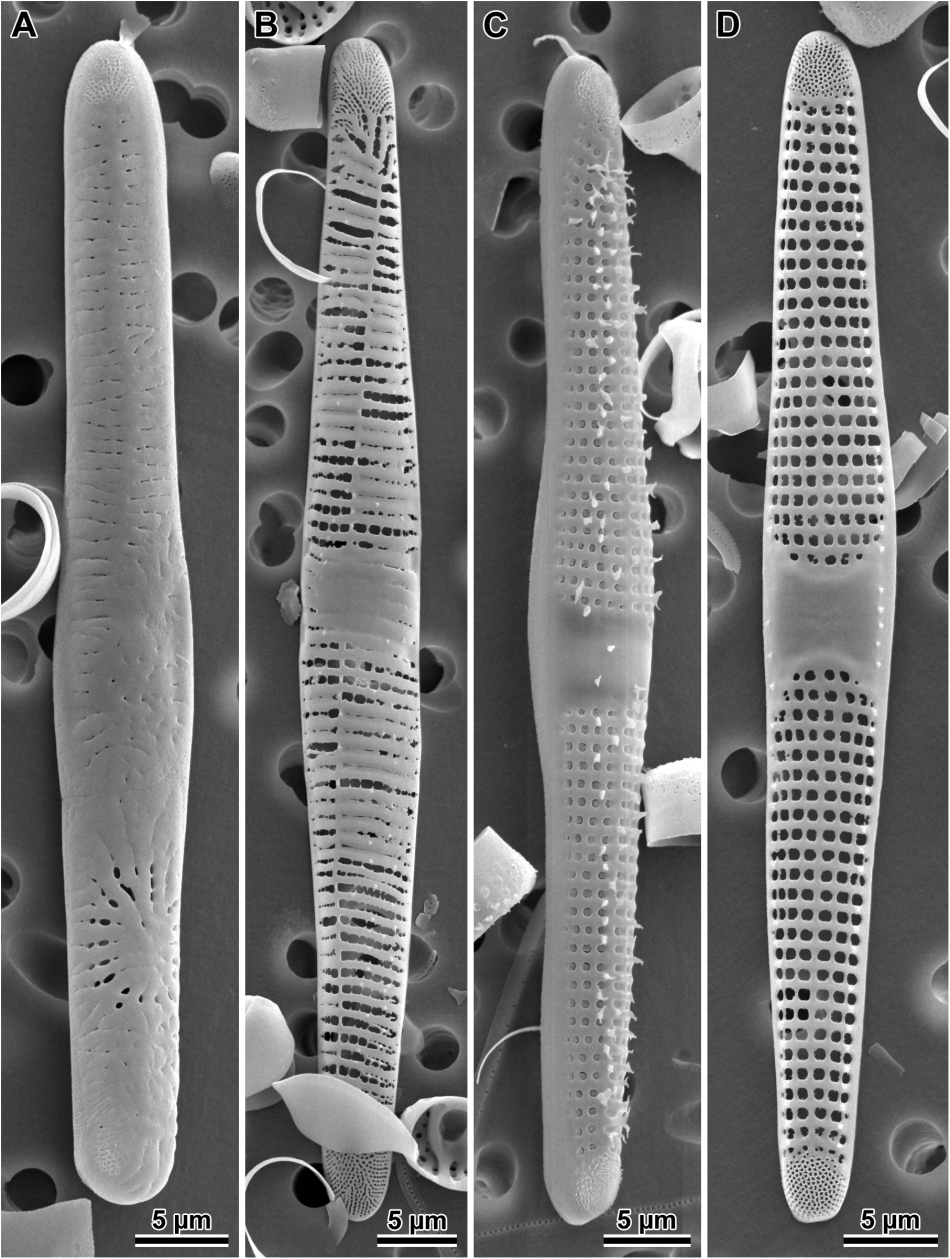
Variations in post-sexual valve morphology of *P*. *staurophorum*. (A) Imperfect initial valve with abnormal striae and areolae but with apical pore field. (B) Fairly well developed initial valve with recognizable striae, areolae and pore fields, albeit disorganized striae. (C) Well developed valve with proper striation but disorganized spines. (D) Perfect vegetative valve with only the bases of marginal spines yet developed.

Initial epivalves (Figs 4H, 4I, 4J, 4K, 4L, 4M, 6A, 6B, 6F and 6G) were relatively lightly silicified (Fig 7B, 7C, 7D, 7E, 7F and 7G), shallow, with an ill-defined and poorly developed mantle. These valves were irregularly striated by rows of simple, small pores (Fig 6A, 6B, 6F and 6G), rather than areolae covered by rota. Slightly elevated apices (where apical pore fields are located in perfect valves) were detectable on some initial epivalves. Initial epivalves were associated with at least two ornamented copulae (Fig 6C, 6D, 6E and 6G). The initial epitheca cradled the initial hypotheca in the same manner as a normal vegetative valve. The initial hypovalve had ornamentation readily recognizable as our *Plagiogramma* or *Dimeregramma* species. Heterovalvar frustules containing an initial epitheca and initial hypotheca (Fig 6E) were not found outside auxospore walls. At least one of the copulae of a functional initial frustule (found outside auxospore walls) was a closed hoop (Fig 7A). The initial hypovalve and the consecutive post-sexual thecae were relatively heavily silicified (Fig 7F and 7G).

Somewhat imperfect post-sexual valves, still recognisable as one of our *Dimeregramma* or *Plagiogramma* species, contained a number of familiar structural characters (Fig 8), e.g., pore fields and striae areolae with rota. Structural imperfections/differences on post-sexual valves included ornamented valve copulae, parallel rather than lanceolate valve outline (*D*. *acutumontgo*; see below), slightly sinusoidal or asymmetrical valves, disorganized striae and their areolae, ill-developed fascia (*Plagiogramma* spp.), and valve faces densely “peppered” with spines (Figs 7F and 8C). There must have been only a few such ill-formed valves following initial epivalve deposition, because they were infrequently encountered in the population of post-sexual cells that had undergone a few rounds of mitotic divisions.

### Sexual interactions between our clones and species

Clones from three of our four species regularly sexualised, allowing examination of interactions between them (Table 3). Clones of *Dimeregramma acutumontgo* and *Plagiogramma staurophorum* were unisexual and demonstrated a heterothallic mating system, although the propensity of auxospores resulting from any mating varied depending on the pair. Among *P*. *tsawwassen*, there were heterothallically mating unisexual and polysexual clones and clones auxosporulating intraclonally. The latter term is used throughout the manuscript with respect to these clones because we did not determine whether intraclonal auxospores were produced allo-, auto- or apogamically.

**Table 3:** Pairwise mating results in *Plagiogramma* species.

Species	Clone/Sex		St.A:2	St.A:3	St.A:6	St.A:7	St.A:8	HK212	Van3:10	Van4:1	Van4:3	Van4:5	Van4:7	Van4:8	Van4:11	Van4:12	Van5:1	Van5:2	Van5:4	Van5:5	Van5:6	Van5:12
			M	M	UN	M	F	UN	IN	M	UN	IN	PL	PL	UN	IN	F	M	IN	F	M	PL
*P*. *staurophorum*	St.A:2	M	*																			
	St.A:3	M	*	*																		
	St.A:6	UN	*	*	*																	
	St.A:7	M	*	*	*	*																
	St.A:8	F	G	A	G	A	*															
*P*. *staurophorum sensu* [55]	HK212	UN	-	-	-	*	*	-														
*P*. *tsawwassen*	Van3:10	IN	-	-	-	-	*	-	A													
	Van4:1	M	-	*	-	-	-	-	-	G												
	Van4:3	UN	*	-	*	*	*	-	*	-	*											
	Van4:5	IN	-	G	-	-	-	-	-	A	*	A										
	Van4:7	PL	-	G	-	*	-	-	-	A	-	A	G									
	Van4:8	PL	-	G	-	*	-	-	-	A	*	*	G	*								
	Van4:11	UN	-	-	-	-	-	-	-	-	-	-	-	-	*							
	Van4:12	IN	-	*	-	-	*	-	G	*	*	-	A	-	-	A						
	Van5:1	F	-	*	-	-	*	-	A	A	*	G	A	-	-	A	*					
	Van5:2	M	-	-	-	-	*	-	G	-	*	-	A	-	G	A	G	G				
	Van5:4	IN	-	G	-	A	-	-	-	G	*	G	G	-	-	*	A	-	A			
	Van5:5	F	-	*	-	-	*	*	G	A	*	-	A	A	-	A	*	A	A	*		
	Van5:6	M	-	*	-	-	*	*	*	*	*	-	*	A	-	A	A	*	A	A	*	
	Van5:12	PL	-	-	-	-	-	-	*	-	-	-	-	-	-	*	G	-	*	A	A	*

* = no sexual products from mating; G = gametes produced; A = auxospores produced;— = no mating performed; F = female; M = male

UN = sex undetermined; IN = intraclonal auxosporulation; PL = auxosporulates in presence of both females and males

Interspecific pair mixtures produced no auxospores, except in one instance involving the *P*. *staurophorum* heterothallic male clone StA:7. However, in this one case, the auxosporulation could also be explained by the other clone-member of the pair (*P*. *tsawwassen* Van5:4; Table 3) engaging in intraclonal auxosporulation rather than by interspecific crossing. Three other clones of *P*. *tsawwassen* (Van4:5, Van4:7, Van4:8, intraclonal and polysexual clones respectively; Table 3) mixed with another *P*. *staurophorum* male clone (StA:3) resulted only in gametogenesis.

Intraspecific sexual interactions were more complicated between clones of *P*. *tsawwassen*. One clone never sexualised, despite numerous mating-trials with nearly all clone/species combinations (Van4:3; Table 3) conducted over several years, and despite having 18S and *rbc*L sequences identical to clones that did sexualise, in the fragments we examined. About half of the inducible clones maintained a mostly unisexual nature and mated heterothallically. Four (out of 14) clones auxosporulated intraclonally, while an additional three (Van4:1 and Van5:2) produced only gametes in their control wells (Table 3). Sex expression in intraclonally auxosporulating clones and in their combination with any other clones is not considered any further here because it requires a separate study to determine the parentage of initial cells.

Results of mating unisexual clones of known sex suggest the existence of polysexual interactions among some of *P*. *tsawwassen* clones. For example, the individuals of Van4:1 produced only gametes in control wells, but auxospores developed in the presence of female (Van5:1 and Van5:5; Table 3), not male clones. Another clone (Van4:7) also produced gametes in control wells, but auxospores were produced only when it was mixed with either unisexual male (Van4:1 or Van5:2) or female clones (Van5:1 or Van5:5). However, another unisexual clone (male Van5:6) did not elicit any sexual response from Van4:7 (Table 3). Similarly, complex interactions took place between clones Van4:8 and Van5:12 with the unisexual clones of both genders, but in mating with some other females only gametes were produced (Table 3).

Finally, we observed that some clones retained their capacity to sexualise (Fig 8B and 8C; small valves represent parental valves) even after five years in culture resulting in dramatic diminution of their cell size (Table 2), while others did not. When one member of a pair was tagged with PDMPO to distinguish it from its mating partner of the same cell-size in 2016, *P*. *staurophorum* (males StA:2, StA:7; female StA:8) and *P*. *tsawwassen* (female Van5:1; males Van5:2 and Van5:6) demonstrated the same phenotypic sex expression as in 2010–2011.

### Phylogenetic analyses (Figs 9 and 10)

**Fig 9:**
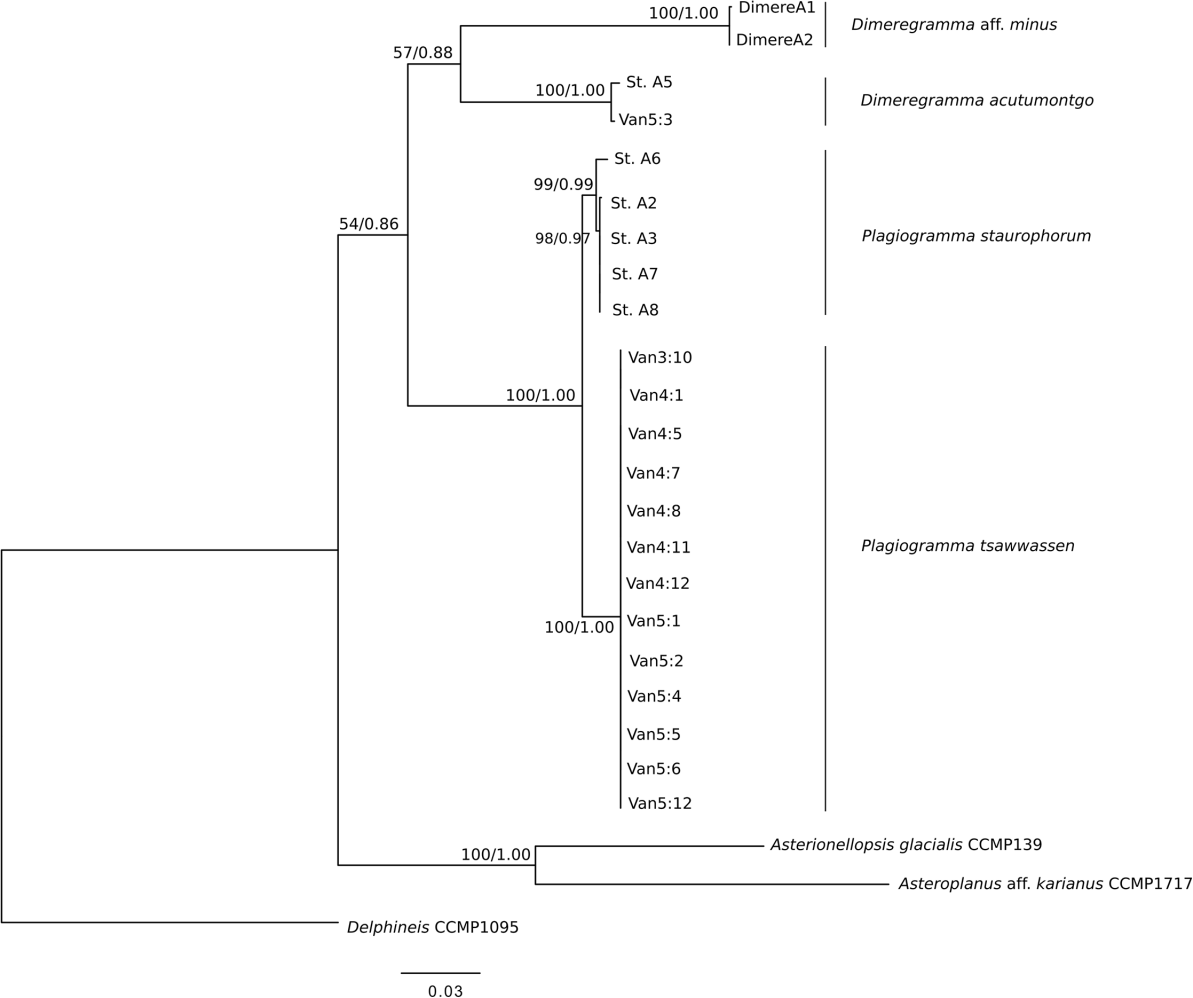
Phylogeny of 22 Plagiogrammaceae strains from this study. Maximum Likelihood tree inferred using RAxML v. 8.2.0 with three concatenated markers, SSU rDNA, ITS, and *rbc*L. Outgroups are *Delphineis* CCMP 1095, *Asterionellopsis* cf. *glacialis* CCMP139 and *Asteroplanus* aff. *karianus* CCMP1717 (reported as *A*. *socialis* ECT3920 in [32]). Values above or below nodes show Maximum Likelihood bootstrap values > 500 (out of 1000) as percentages, and Bayesian posterior probabilities. Scale bar shows number of substitutions per position.

Phylogenetic analysis of the three gene markers (18S, ITS and *rbc*L) retrieved trees confirming monophyly and identity of all four species examined. These trees had similar overall topology, with two exceptions: the 18S tree could not separate clades of *Plagiogramma* (S1A Fig), and the ITS tree recovered *Dimeregramma* as a paraphyletic clade (S1C Fig), with our Caribbean strains forming a cluster with *Delphineis*. The other two markers recovered *Dimeregramma* as monophyletic with strong bootstrap support (S1A and S1B Fig). The tree of all three concatenated gene markers preserved the monophyly of both *Plagiogramma* and *Dimeregramma* as analysed by ML and Bayesian analysis (Fig 9 and S2A Fig). The unexpected results in the ITS phylogeny may be due to strong divergence in this gene, insufficient taxon sampling, and/or an outgroup that was too distant. In the future, the resolution may be improved by the addition of more related ITS sequences to reference databases.

**Fig 10:**
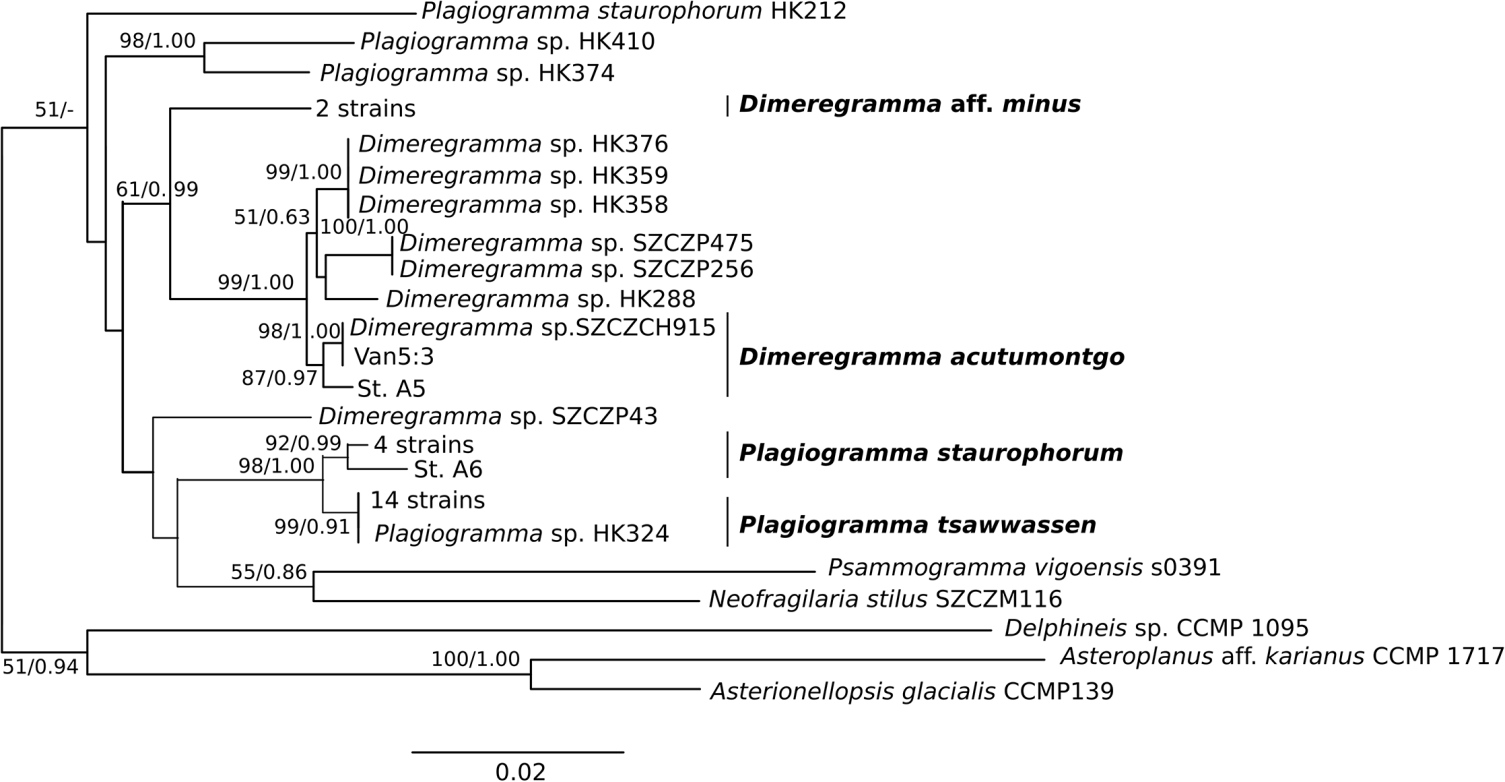
Phylogeny of 37 Plagiogrammaceae strains and additional sequences from the study of [32]. Maximum Likelihood tree inferred using RAxML v. 8.2.0 with two concatenated markers, SSU rDNA and *rbc*L. Outgroups were: *Asterionellopsis* cf. *glacialis* CCMP139 and *Asteroplanus* aff. *karianus* CCMP 1717 (reported as *A*. *socialis* ECT3920 in [32]), and *Delphineis* sp. CCMP 1095; taxonomic justification for species names in [9]. Values above or below nodes show Maximum Likelihood bootstrap values > 500 (out of 1000) as percentages, and Bayesian posterior probabilities. Scale bar shows number of substitutions per position. Names of strains from this study are given in full in Table 1.

A two-marker phylogeny (18S and *rbc*L) of our strains was compared to the four-marker phylogeny of [32] (18S and *rbc*L, plus 28S rDNA and *psb*C), and recovered a similar topology (Fig 10), except that a few of their strains could not be resolved to genus-level in our tree: HK410, HK374 and SZCZP43.

Our analysis shows that their strain HK324 had 100% sequence identity for these markers with our *Plagiogramma tsawwassen* clones, while the strain SZCZH915 had 100% sequence identity with one clone of *Dimeregramma acutumontgo*. HK212, named *P*. *staurophorum* in [32], but found here to be *Plagiogramma* aff. *staurophorum*, branched with low bootstrap support from the base of all other plagiogrammacean strains, and may represent a species new to science (see taxonomic discussion below). While we recovered good support in both ML and Bayesian analyses for clades containing clones from individual species examined in this study, the higher groupings are not well supported and differed between the two methods of analysis (Fig 10 and S2B Fig). We therefore consider the branching order above species level to be unreliable using these markers.

### Taxon identity and characterization

#### *Dimeregramma acutumontgo* B. S. Gray Jr. and Kaczmarska, species nova (Fig 11)

Synonym: *Dimeregramma acutum sensu* [56] (Plate 97 fig. B) and [9]

**Fig 11:**
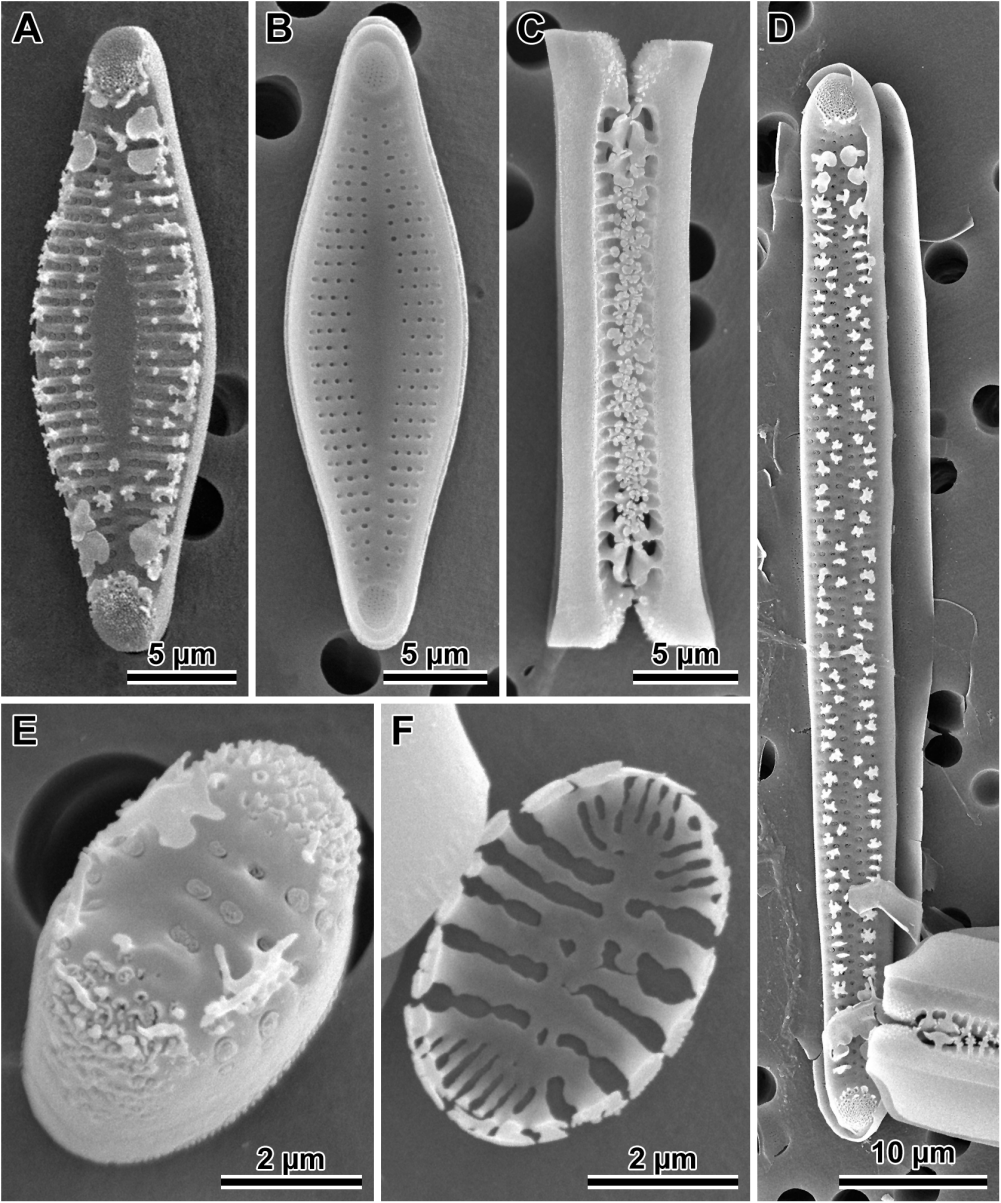
*Dimeregramma acutumontgo*. (A) External and (B) internal view of a typical vegetative valve from the holotype preparation No. B 40 0042012 at BGBM, on SEM stub 194–16. (C) Girdle view of two sibling valves showing colony formation from the same preparation. (D) External view of a post-sexual valve face of a progeny of StA:5 mated to Van5:3. (E) External view of a valve face of a StA:5 specimen after 6 years in culture. (F) Internal view of a very small valve from the same clone taken in 2015–2016, showing cell-size related culture induced morphological modifications.

Holotype: DMF SEM stub 194–16 deposited at Botanischer Garten und Botanisches Museum, Berlin (BGBM) as preparation No. B 40 0042012 and illustrated in Fig 11A; in culture as clone StA:5; fixed, non-cleaned material from that cultured clone as B 40 0042013 at BGBM. Differential interference contrast (DIC) light photomicrographs of the valves are shown in S3A and S3B Fig. Sequence fragments of *rbc*L, SSU rDNA gene and the ITS region are deposited in GenBank and the BOLD System (Table 1).

Type locality: intertidal mudflat at Indian Point, St. Andrews, New Brunswick Canada; geographic coordinates given in Table 1.

Description: Cells joined by branching interdigitating spines along the sibling valve margins (Fig 11A, 11C and 11E), forming short to medium length chains dispersed in one clone (StA:5) but clumped together in the other (Van5:3); valve face not flat in girdle view due to two sub-apical depressions and three valve surface elevations (two apical and one central); the valve face outline from nearly linear (post-sexual) through widely lanceolate (midsize specimens; Fig 11A and 11B) to ellipsoidal (in very short specimens; Fig 11E and 11F), with protracted apices in longer valves, and rounded-conical in shorter valves. In near-natural specimens (those examined soon after establishing the clone in 2010–2011, before cell diminution and culture induced modification of valve morphology took place; Table 2), and in post-sexual cells, apical valve length ranged from 23–72 μm, transapical length ranged from 5.2–9.5 μm (Table 2). Externally, apical pore fields in regularly arranged rows and/or rings of pores occupy most of the apical elevations; papillae and occasionally large projections border these fields (Fig 11A, 11D and 11E). Sternum clearly defined, lanceolate, and widest in valve mid-section in longer specimens (Fig 11A and 11B), indistinct in post-sexual and parallel in small, elliptical valves (Fig 11E and 11F). These morphologies occur within the same clone examined over six years of the clone life span. Striae uniseriate, 12–14 in 10 μm with round or elliptical areolae, covered by rota, 13–19 in 10 μm. The mantle distinct, non-perforated, begins below the row of marginal spines; one spine stands at nearly every interstriae. However, our 2016 specimens of clone StA:5 after six years in culture, which were smaller than any of the diagnostic descriptions for a species of this genus, show areolae on the mantle, in contrast to specimens of the same clone when their cells were of greater size (compare Fig 11E with Fig 11B and 11C). An additional row of smaller and simpler spines outlines the wider section of the sternum in strongly silicified, completely developed valves (Fig 11A). Spines dispersed throughout the face in post-sexual valves (Fig 11D). Distal ends of the marginal spines modified into ear-shaped projections interdigitating between sibling valves holding frustules in chains. The ends of the 1–2 subapical pairs modified into “flaps” bent parallel to the valve face surface (detectable only in SEM; Fig 11A, 11C, 11D and 11E), each facing a similar “flap” on spines of the sibling valve.

Internal valve view shows sternum and uniseriate striae separated by wide interstriae (Fig 11B); striae areolae deep, with rota. Rimoportulae absent (Fig 11B). Apical pore-fields located in circular-to-elliptical depressions corresponding to external apical elevations. Valvocopulae are wide and plain (Fig 11C); one row of pores present on the other, narrower copulae. Differential interference contrast (DIC) light photomicrographs of the valves are shown in S3A and S3B Fig.

**Fig 12:**
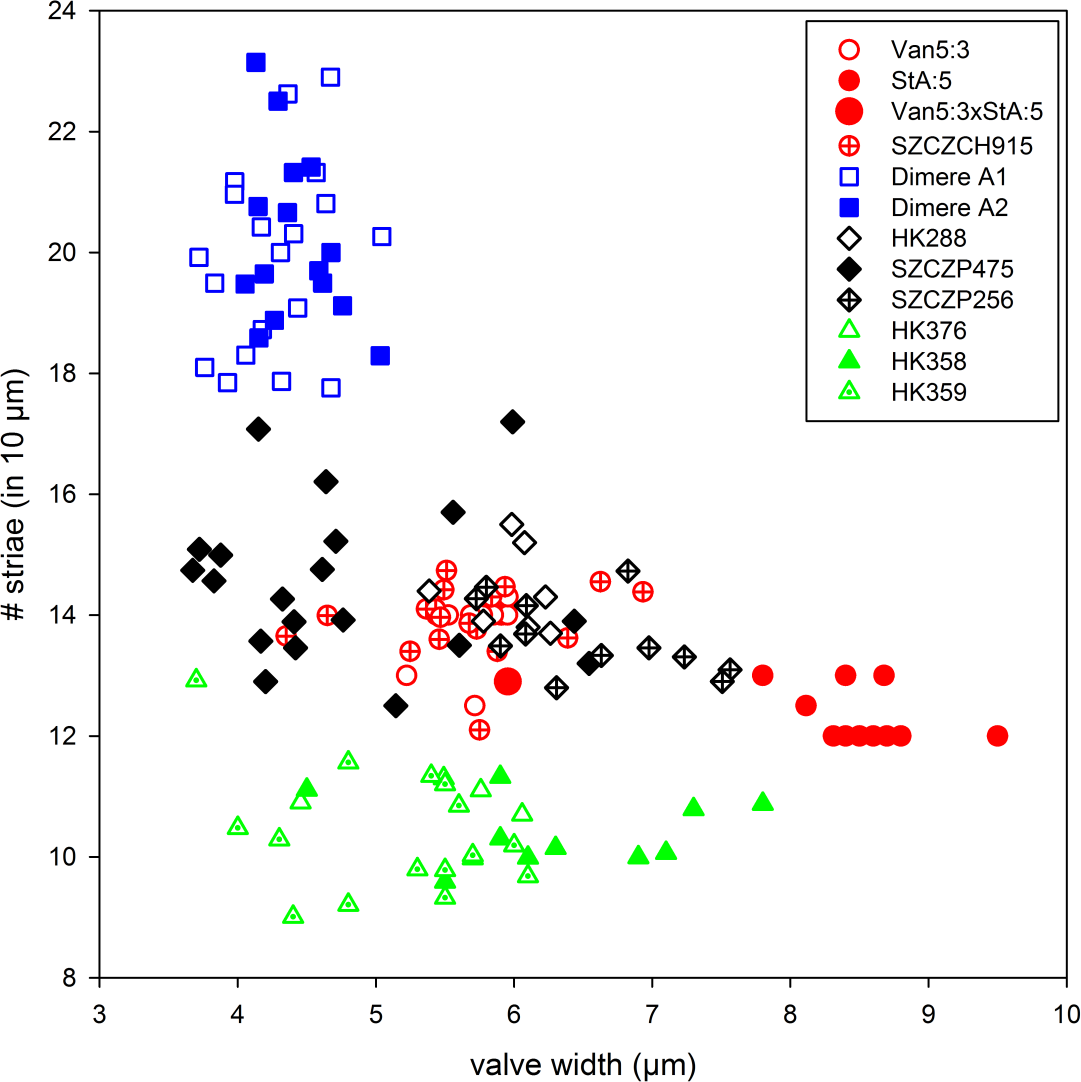
Scatter plot of *Dimeregramma* clones from this study and [32], illustrating the relationship between valve width and striae density. Red symbols indicate *D*. *acutumontgo* soon after clone establishment, their sexual progeny, and the clone SZCZCH915; black (clones SZCZP474, 256 and HK288) and green symbols (HK358, 359, 376) indicate six exotic clones from [32] which constitute a sister clade to *D*. *acutumontgo* in Fig 10; blue symbols indicate *Dimeregramma* aff. *minus*.

Valve width and number of striae (illustrated in Fig 12) are the least variable characters of valve morphology among those examined here. These two characters are likely the most useful in species identification, particularly in natural samples.

Diagnosis: Morphological signature: sternum lanceolate in outline in lanceolate valves, parallel, irregular or absent in smallest, elliptical valves while indistinct in post-sexual valves; striae fine, 12–14 in 10 μm, uniseriate, 13–19 areolae in 10 μm; spines on valve margin and along sternum margin, best evident in fully silicified valves.

Molecular signature: consists of two nuclear encoded DNA fragments: a conserved section of the SSU rDNA gene and ITS region, and a plastidal DNA fragment from the *rbc*L gene; accession numbers in Table 1.

Comparison to similar congeners: *Dimeregramma* is a relatively small, but insufficiently researched genus established by Ralfs (in [57], p. 790) to contain already known taxa attributed mostly to the genera *Denticula* and *Odontidium*. Ralfs’ generic delineation is quite broad, thus a number of species previously attributed to this genus have been transferred to other new or redefined genera, e.g., *Talaroneis*, *Pteroncola*, *Hyaloneis*, and others.

Currently *Dimeregramma* comprises approximately two dozen valid extinct and extant species [58]. Taking into account valve and sternum outlines, they may be segregated into two groups: one with sternum outlines more or less linear (e.g., *D*. *angustatum* Hajos, *D*. *dubium* Grunow, *D*. *fusiformis* Huang, Cheng et Chin, or *D*. *intermedium* Boyer; Table 4) and valve outlines with or without inflations or constrictions; the other with species having lanceolate-elliptical valve outlines and a lanceolate sternum (e.g., *D*. *acutum sensu* Montgomery, *D*. *minus* (Greg.) Ralfs, or *D*. *scutellum* Hanna; Table 4). Our mid-size specimens, examined soon after clone establishment and thus considered to represent nearly natural specimens, have a lanceolate valve outline and the sternum widening in the valve mid-section but have striae and/or striae areolae finer than any congener for which these metrics are available (Table 4).

**Table 4:** Morphological summary of vegetative valves of the best-documented species of the genus *Dimeregramma*.

Species	Source	Valve Shape	Sternum Shape	Length (μm)	Width (μm)	Striae (in 10 μm)	Pores (in 10 μm)
*D*. *acutumontgo* sp. nov.	2011*	lanceolate	narrowly lanceolate	23–72	5–9.5	12–14	13–19
	CH915*	lanceolate	narrowly lanceolate	19–24	5.2–5.9	12–14	15–19
*D*. *acutum* Hust. (*sensu* Montgomery)	[56]*	lanceolate	narrowly lanceolate	28.8–35.5	7–9	11–12	14 (~tilt)
*D*. *acutum* Hust. (t.m.)	[59]	narrow lanceolate	indistinct linear	16.0	4.0	20.0	unresolved
*D*. *lanceolatum* Peragallo (t.m.)	[60]	linear-lanceolate	lanceolate	30.0	10	6	3 per stria
*D*. *maculatum* (Cleve) Frenguelli	[59]	widely lanceolate	widely lanceolate, fascia	55–50	8–15	9–15	na
*D*. *minus* (Greg.) Ralfs	[57]	narrowly lanceolate	na	na	na	7–8	na
(= *Denticula minor* Greg.; t.m.)	[61]	lanceolate-fusiform	lanceolate	13–51	5–15	7–9	na
	[62]	lanceolate	lanceolate	20–40	6–10	9–10	na
	[56]*	lanceolate	narrowly lanceolate	12–32	6.7–15	6–12	8
	[63]	lanceolate	lanceolate	16–18	8–9	9–10	10
	[64]*	lanceolate	lanceolate	18–20	8	8–9	10–11
*Dimeregramma* aff. *minus*	[50]*	elliptical	narrowly parallel	5.5–8	3.5–5	14–25	18–26
*D*. *minor* var. *nana* (Greg.) Van Heurck	[65]*	linear-elliptical	indistinct, linear	9.3–11	3.5–4.5	12–14	2–3 per stria
(= *Denticula nana* Greg.; t.m.)	[61]	rhombic	linear	12.5–25.3	na	rather fine	na
	[64]*	rhombic-lanceolate	rhombic-lanceolate	18	6.2	14	na
	[62]	lanceolate	lanceolate	10–20	6–10	14	2–3 per stria
	[66]	rhombic	rhombic-lanceolate	10–20	na	14	na
	[67]*	rhombic-lanceolate	rhombic-lanceolate	19–31	12	12	~12
*D*. *scutulum* Hanna	[68]	rhombic-lanceolate	rhombic-lanceolate	33.0	13.3	9.0	3+ per stria
*D*. *angustatum* Hajos	[69]*	linear, round cone ends	indistinct linear	87.0	9.0	5–6	11.0
*D*. *costatum* Peragallo	[60]	lanceolate	linear-lanceolate	20–25	7–8	5–6 ribs	1.0
*D*. *boryanum* Pantocsek	[70]	linear	na	45.0	8.0	15.0	15.0
*D*. *fluens* Mann	[71]	linear	widely lanceolate	105.0	13.0	7.0	na
*D*. *fusiformis* Huang, Cheng et Chin	[72]	fusiform	indistinct linear	13–20	3–6	27–28	27–28
*D*. *fulvum* (Greg.) Ralfs	[62]	linear-lanceolate	indistinct linear	30–110	6–10	10–11	na
*D*. *inflatum* Mann	[73]	8X longer than wide	linear, center widened	86.0	15.0	na	na
*D*. *intermedium* Boyer	[74]	rhombic-lanceolate	indistinct linear	35.0	na	10.0	na
*D*. *lapponica* A. Cleve	[66]	lanceolate, mid constricted	narrow linear	77.0	10.0	18.0	na
*D*. *marinum* (Greg.) Ralfs	[62]	linear, central swelling	linear, central swelling	50–220	6–10	5–7	2–3 per stria
(= *Denticula marina* Greg.)	[60]	linear, central swelling	narrowly linear, central swelling	80–150	na	4–6	na
	[63]	linear, central swelling	linear, central swelling	60–80	7.5–15	7–8	6.7–8
*D*. *opulens* Mann	[71]	linear, biundulated	indistinct linear	56–79	14–17	5.5	alveolae
*D*. *rostratum* Hustedt	[59]	linear	indistinct linear	15–17	4.0	~18	~18
*D*. *tiltilense* Frenguelli	[75]	centrally inflated	very narrow	51–62	14–15	13.0	na
*D*. *tortonicum* Hajos	[69]*	lanceolate	narrowly linear	28–32	12.0	6–7	5–6
*D*. *ventricosum* Janisch & Rabenhorst	[76]	centrally inflated	indistinct linear	na	na	na	na

* = determined using SEM; na = not available; t.m. = type material

Our *D*. *acutumontgo* specimens demonstrate some of the characters known in two taxa: *D*. *acutum sensu* [56] and *D*. *minus* (Greg.) Ralfs [61]; the latter will be discussed further below. Montgomery [56] was apparently the first to examine these diatoms using SEM, and called his specimens *D*. *acutum*, even though the valve striae in his specimens were coarser than Hustedt’s specimens (Table 4). In addition to regular marginal spines, the Florida valves carried pronounced marginal “flaps”, such as we observe on our specimens (Fig 11A, 11C and 11D). Whether such “flaps” occur on *D*. *acutum* (*sensu* [59]) remains to be determined, because despite being present, they were undetectable in LM preparations of our own specimens. They were also undetectable on Hustedt’s original specimens illustrated by [77].

*Dimeregramma acutum* Hustedt was described from the Atlantic coast of North Carolina, USA as a small diatom with narrowly lanceolate valves (Table 4) and acutely rounded ends illustrated in [77] (Plate 602, figs. 14–16). In agreement with *D*. *acutum* Hustedt, our new species valve outlines are lanceolate-fusiform, including somewhat acute apices and similar cell-size, but they differ from *D*. *acutum* Hustedt in having coarser striation, and a lanceolate rather than parallel (in Hustedt’s species) sternum.

In comparison, *Dimeregramma minus* (Greg.) Ralfs shares the lanceolate shape of the sternum in larger valves of our specimens, but it is more coarsely striated in the original species delineation and most of the major monographs with the exception of [66, 78].

Seven unnamed clones attributed to the genus *Dimeregramma* (SZCZCH915, SZCZP256 and 475 together with HK288, 358, 359, and 376) reported by [32] show considerable genetic and morphological similarity to our new species (Figs 10 and 12, Table 2) in the characters considered here. Of these, SZCZPH915 is the most similar genetically. This clone, together with our two Canadian clones form a strongly supported clade, which is a sister to the clade containing all six other clones (Fig 10), albeit with weak support. Morphologically, *Dimeregramma* sp. SZCZPH915 is indistinguishable from our Pacific clone Van5:3 and its progeny with Atlantic StA:5 in their least variable characters (Fig 12). Of the six clone members of the poorly supported sister clade, three unnamed clones of *Dimeregramma* spp. (HK288, SZCZP256, 475; Fig 12) are also morphologically inseparable from *D*. *acutumontgo* (Fig 12). In contrast, the three other clones (*Dimeregramma* sp. HK358, 359 and 376) group together as a largely separate, somewhat coarsely striated valve cluster (Fig 12). It is likely that the Korean clone SZCZCH915 belongs to our new species. However, unravelling the identity of the remaining six clones of *Dimeregramma* spp. from [32] will require separate study that is beyond the scope of this manuscript. Nonetheless, the conclusion emerging from all the data at hand points to the existence of one or two additional new species morphologically similar but genetically distinct from our *D*. *acutumontgo*.

Distribution: Two sexually compatible clones were collected, one each from Canadian Atlantic and Pacific coasts (Table 1). Morphologically similar specimens are also reported from coral reefs of the Florida Keys, USA as *D*. *acutum* [56]). Genetically and morphologically similar clones of unnamed species belonging to the genus *Dimeregramma*, as currently defined, were also reported from the Floridian and Texan coasts of the Gulf of Mexico (HK288, HK358, and HK359; [32], Suppl. fig. 3f, c), South Carolina, USA (HK376; [32], Suppl. fig. 3d), Korea, South Africa and Namibia (SZCZCH915, SZCZP475 and SZCZP256, respectively; [32], Suppl. fig. 3g, e). Even if only our clones and the Korean clone (SZCZCH915) specimens represent the same species, the diatom is widely distributed in both northern Atlantic and Pacific Oceans.

#### *Dimeregramma* aff. *minus* (Greg.) Ralfs (Fig 13)

Synonym: *Dimeregramma minor* var. *nanum sensu* [50]

**Fig 13:**
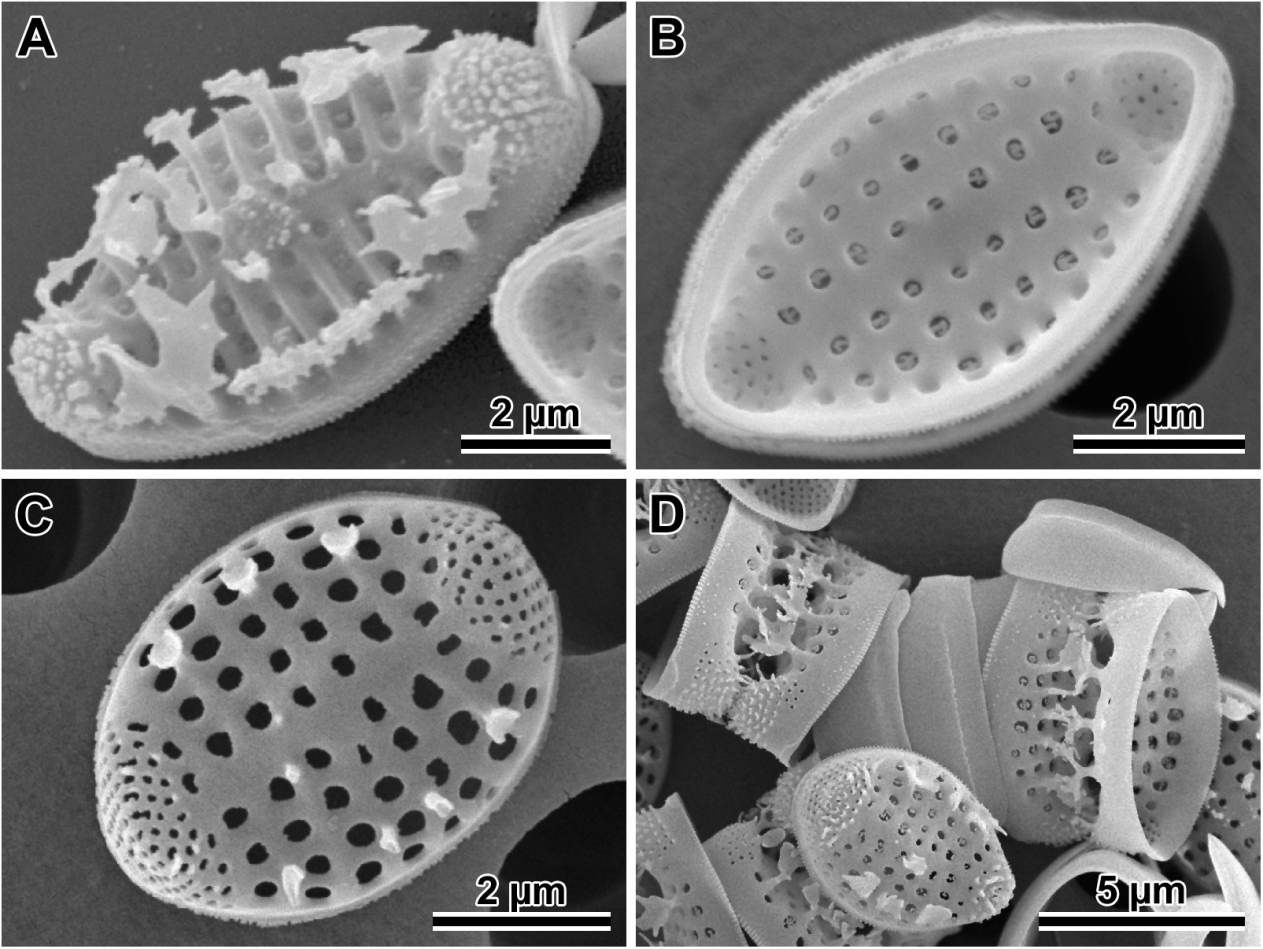
*Dimeregramma* aff. *minus*. (A) External and (B) internal view of the valve face of strongly silicified specimens from preparation No. B 40 0042014 at BGBM, on SEM stub 197–13. (C) External view of weakly silicified valve with incompletely formed spines around apical pore fields, marginal and near-sternum located spines. (D) Several sibling valves illustrate mode of colony formation and valvocopulae.

(NOTE: *the correct epithet for the species is “*minus*” because the gender of the new genus* Dimeregramma *Ralfs to which the species was transferred is neutrum*, *while* Denticula, *to which Gregory initially attributed the species*, *is femininum; Drs*. *R*. *Jahn and H*. *Kusber*, *personal comm*., *2015*)

Morphological signature: chain forming species (Fig 13A and 13D). In girdle view, valve face shows three elevations; the central elevation often with a ring of short and/or a few larger spines scattered about (Fig 13A). Valve face outline is rhombic (Fig 13B) with conical apices; valves are 5.6–7.9 μm long and 3.7–5.0 μm wide. Externally, apical pore fields have radially arranged rows of pores (Fig 13A and 13C); spines, papillae and occasionally large projections intermix with pores and border these fields (Fig 13A and 13D). Sternum narrow and linear but distinct in all valves with normal morphology (Fig 13B and 13C) and rhombic valve outline; short spines dispersed along the sternum (Fig 13A). Striae uniseriate, 18–23 in 10 μm; striae areolae round-to-elliptical, covered by rota, 18–26 in 10 μm (Fig 13B) located between thick, elevated interstriae; clone morphometrics are given in Table 2. Valve mantle is distinct, beginning below the row of marginal spines and has at least one row of areolae. One marginal spine located on every 1–2 interstriae (Fig 13D); distal ends of these spines carry branching and/or ear-shaped projections interdigitating between sibling valves to hold frustules in chains. The ends of 1–2 pairs of subapical spines in form of a solid flap that is parallel to the valve face (noticeable in SEM), each facing a similar spine-flap structure on the sibling valve (Fig 13A and 13D).

Internal valve structure displays a clear linear sternum and uniseriate striae separated by wide interstriae. Apical pore-fields located in an area of apical depression (Fig 13B) corresponding to external apical elevations; rimoportulae absent. Valvocopulae wide and plain; one row of pores is present on the other, narrower copulae. Nuclear and plastidal markers of the clones attributed to this taxon can be found in Table 1. Our two clones available did not sexualise despite repeated attempts.

Comparison to similar congeners: Our specimens are most similar to *D*. *minus* var. *nanum* in valve size and outline, although they are even smaller and more finely striated than metrics reported for this taxon (Table 4). Similarly, the proximal end of the last pair of marginal spines is also flattened to form “flaps” ([27], fig. h, and possibly fig. e; in presumably *D*. *minor*, the generitype demonstrated in [27], p. 242). Our specimens differ from this and other varieties recognized within *D*. *minus* by having a linear rather than rhombic sternum and much finer striae (Table 4 and Fig 12). Two other species with some morphological affinity to our specimens are *D*. *dubium* and *D*. *rostratum*. The former is similar to our specimens in having somewhat conical valve ends, but differs by having coarser striation. The latter has striae density similar to our specimens, but differs by having distinctly rostrate valve apices. Table 4 summarizes available information for some twenty morpho-species best documented in published sources, and indicates that our specimens do not conform well to any of them.

The extremely small size of our cultured cells suggests that they may be near the minimal end of the species-specific cell-size range. Thus, they may represent atypical species morphology. For example, the comparison of metric characters in typical vegetative cells of e.g., *D*. *acutumontgo* (Table 2; cells of clone StA:5 measured in 2016) or post-sexual cells of *Plagiogramma staurophorum* to those that are very small (Table 2) illustrates the variability in valve morphology with decrease in size. For these reasons, despite its morphological distinctiveness (striation finer than any reported in this genus, save *D*. *fusiformis*; Table 4), we refrain from proposing this diatom as a species new to science. Molecular characterization (accession numbers in Table 1) of the two clones provided here should help to recognize larger-cell conspecifics with typical morphology, and ultimately will contribute to unravelling their true taxonomic identity.

#### *Plagiogramma staurophorum* (Gregory) Heiberg (Fig 14)

Original material examined: Three preparations curated in the British Museum of Natural History (BM-1205, BM-1418, and BM-1422, gathered originally at Loch Fine and Arran [61]) were examined using LM; unprocessed original material is not available.

**Fig 14:**
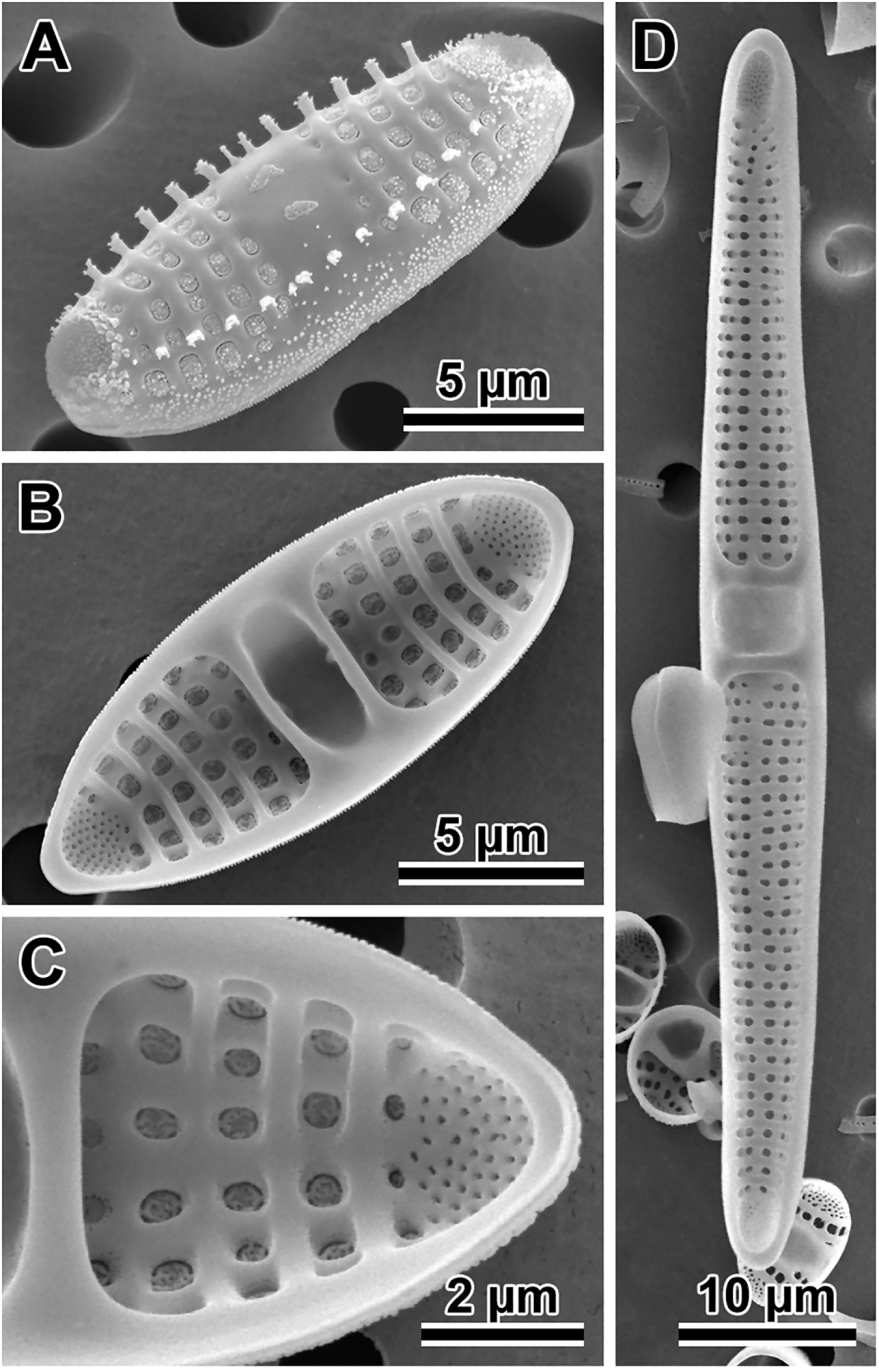
Plagiogramma staurophorum. (A) External and (B) internal view of the valve face. (C) Close up of the interior of the valve. (D) Internal view of the valve of a sexual progeny from mating of clones StA:7 and StA:8, note difference between parental (three small valves in lower part of the image) and progeny cell size (center specimen).

Morphological signature: specimens from Gregory’s original material examined here had valves 23–45.5 μm long and 5.8–8.8 μm wide, lanceolate when longer but with blunt apices and elliptical valve outline when shorter, marked with punctate striae, except in the valve mid-section, where there is a broad fascia bordered by internal costae. There are 8.5–14 punctate striae in 10 μm; punctae slightly more numerous than striae; sternum very narrow. A specimen from original sample BM1350 can be found in [79] (p. 30, and Plate 15, fig. 98).

Our specimens: They were chain forming diatoms with marginal spines, one spine on nearly every interstria. Valves elliptical-lanceolate (Fig 14A and 14B) in near-natural (those examined soon after establishing the clone in 2010–2011; Table 2) and post-sexual specimens 15–75 μm long, 5.4–10 μm wide; valve face relatively flat and indistinct mantle. The central portion of the valve face forms a fascia (Fig 14A, 14B and 14D) with faint traces of 3–4 rows of silica-filled striae frequently detectable. The fascia often bears distinct granulations concentrated into 1–3 patches. Striae parallel, uniseriate, 7–13 in 10 μm; striae areolae large, covered by reticulate rota, 8–14 areolae in 10 μm counted along striae (Fig 14C) extend below the row of marginal spines; individual clone morphometrics in Table 2. Apical pore fields bordered by small spines and papillae that may be interspaced with the pores. Short marginal spines are located on interstriae at the valve face-mantle junction (Fig 14A); spines are dispersed throughout the valve face in post-sexual cells.

Internally, valve bisected by two transapical costae located at the striae-fascia boundaries (Fig 14B, 14C and 14D); rimoportulae are absent. Copulae open, narrowing towards the apices. Valvocopula is widest, non-perforated; pores present only on other copulae. Nuclear and plastidal markers of the clones attributed to this species can be found in Table 1. DMF SEM stub 195–18 containing valves presented here and illustrated in Fig 14 is deposited as BGBM preparation No. B 40 0042015; in culture as clone StA:8; fixed, non-cleaned material from that cultured clone as BGBM No. B 40 0042016. Differential interference contrast (DIC) light photomicrographs of the valves are shown in S3C and S3D Fig.

Comparison to similar congeners: Gregory's species delineation ([61]; p. 496) calls for frustules that are 25.4–96.5 μm long, 12.5–20.3 μm deep when seen in girdle view, with 5–6 striae in 10 μm. These striae are therefore coarser than reported by the most commonly used diatom monographs and florae (Table 5). They are also coarser than we observed in the original material preserved in British Museum preparations BM 1205, 1418, and 1422, as did [79] in BM1350, which contain specimens of the valves conforming well to Gregory's species delineation in all characters except the striation. We measured twelve consecutive valves from two original preparations and found that all have striation finer than the original description calls for (Table 5). If there are coarser specimens of *P*. *staurophorum* in Loch Fine and Arran samples curated at the British Museum than those we encountered, they are absent in the three preparations we examined.

**Table 5:** Morphological summary of the members of the genus *Plagiogramma* with lanceolate valve face outline, two or no internal costae.

Species	Source	Valve Shape / Costa	Length (μm)	Width (μm)	Striae (in 10 μm)	Pores (in 10 μm)
*Plagiogramma staurophorum* (clones)	this paper*	narrowly lanceolate	15–75	5.5–10	7–13	8–14
*P*. *staurophorum* Greg. (t.m.)	[61]	lanceolate	25–97	na	5–6	~ as striae
*P*. *staurophorum* Greg. (t.m.)	British Museum slides BM1418, BM1422	narrowly lanceolate	22–44	5.8–8.8	9–14	7–9
*P*. *staurophorum* (Greg.) Heiberg	t.m. in [79]*	narrowly lanceolate	30–33	8.5	9–10	7
(= *P*. *gregorianum* Grev.)	[80, 81]	lanceolate	35.5–76	na	7.1	na
	[79]	lanceolate taper (post-sexual)	30–64	4–5.5	9	10
	[66]	elliptical-lanceolate to -linear	12–65	5–11	8–9	10
	[67]*	lanceolate	20–52	5–9	8–10	6–7
	[78]*	lanceolate to linear-elliptical	15–34	5–8	9–12	na
	[62]	linear lanceolate to elliptical	12–65	5–11	8–11	8–11
	[82]	lanceolate to oblong lanceolate	45	na	7–9	na
	[59]	elliptical-linear	34–36	11–13	9–9.5	9–10
*P*. *appendiculatum* Griffen (t.m.)	[83]	rhombo-lanceolate, very wide fascia	10–46	5–6.5	14–15	14–16
*P*. *attenuatum* Cleve	[82]	linear-lanceolate, narrow apices	50	na	10	na
*P*. *decussatum* Grev.	[84, 85]	elliptical-oblong	55.7	18–19	7	na
	[82]	oblong-elliptical	21–55	na	8	na
	[60]	lanceolate-elliptical	20–40	na	9	~ as striae
*P*. *costatum* Grev. (t.m.)	[85]	broadly lanceolate	43.5	11.9	9	6
*P*. *gracile* Hajos (t.m.)	[69]*	narrowly lanceolate	34	7–8	5.5–6	15–18.5
*P*. *jamaicense* Grev. (t.m.)	[85, 86]	girdle view only	38–60.8	na	6.3–12	na
*P*. *laevis* Greg. (t.m.)	[61]	no valve face shown	40–68.6	15	18–19	na
*P*. *laevis* Greg. (as *P*. *leve*)	[62]	lanceolate, weakly capitate	15–65	3–6	14–18	14–18
*P*. *robertsianum* Grev.	[66]	lanceolate	50–65	10	8–9	8–9
	[56]*	lanceolate, rostrate ends	33.2	6.3	12–13	16–18
*P*. *robertsianum* Grev. (t.m.)	[80, 85]	lanceolate, obtuse ends	45.6–76	10–17	11–12	na
*P*. *tsawwassen* sp. nov.	this paper*	narrowly lanceolate	9–32	5–8	13–18	12–20
*P*. *seychellarum* Grun. (t.m.)	[87]	elliptical, NO internal costae	75	13	18 quincunx	~16
*P*. *tenuistriatum* Cleve (t.m.)	[87]	elliptical	32	10	18	na
*P*.? *tesselatum* Grev. (t.m.)	[80, 85]	lanceolate-elliptical, drawn out ends	80–101.3	17.5	3.2–4	na
*P*. *tesselatum* Grev.	[82]	elliptical-lanceolate, NO internal costae	up to 100	na	3	na

* = determined using SEM; na = not available; t.m. = morphology and metrics from type specimen and/or type material

Our specimens conform well to valve outline and all commonly reported metrics for the diatom named *P*. *staurophorum* (Gregory) Heiberg (Table 5). They share some characters with a few other morphologically generally similar species (with lanceolate to elliptical valves with more or less attenuated ends and/or expanded mid-valve and two internal costae) which have been rarely reported since their original description and therefore are documented based only on LM observations, e.g., *P*. *attenuatum*, *P*. *decussatum*, *P*. *costatum*, *P*. *robertsianum*, and *P*. *tenuissimum* (Table 5). Our specimens differ from these in the shape of valve apices (*P*. *attenuatum*, *P*. *laevis*), less regularly organized areolae (*P*. *decussatum*), finer striae (*P*. *costatum*), uniseriate striae structure (*P*. *gracilae*) and coarser striae (*P*. *tenuistriatum*, *P*. *tenuissimum*). *Plagiogramma robertsianum* [80] valve outline, size and density of striae (11–12 in 10 μm) are quite similar to specimens of *P*. *staurophorum* we found in original BM preparations, but would have to be considered a later synonym if proven conspecific.

**Fig 15:**
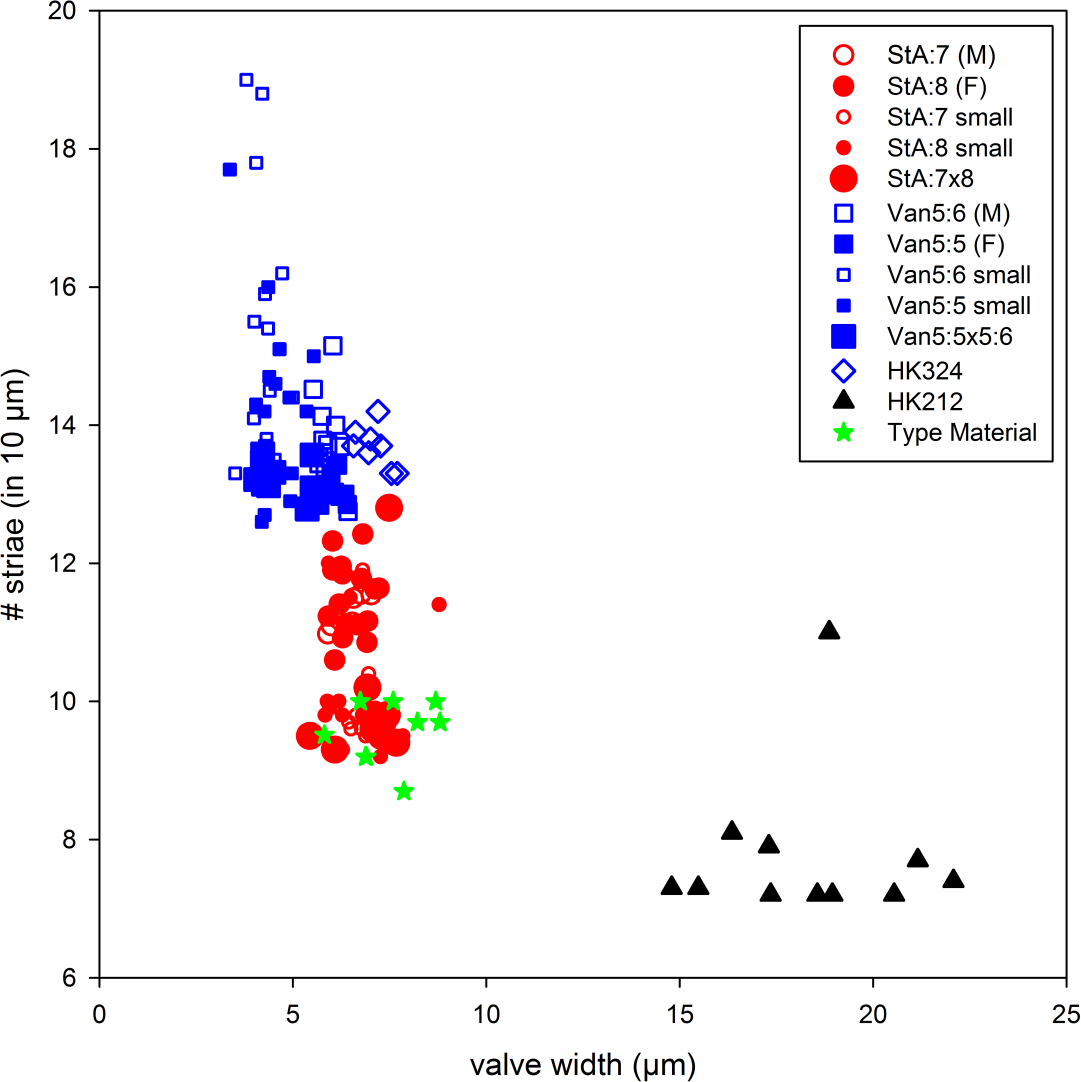
Scatter plot of *Plagiogramma* species illustrating relationship between valve width and striae density. The most sexually active pair of each species of *Plagiogramma* from this study soon after clone establishment (medium-size symbols), after 6 years in culture (named “small”; smallest symbols), and progeny specimens from this pair mating (largest symbols). Red indicates *P*. *staurophorum*, blue indicates *P*. *tsawwassen*, black indicates clone HK212 from [32]. Note green stars, indicating specimens from Gregory's type material for *P*. *staurophorum* measured in brightfield LM, are well integrated into a cluster of our clones of *P*. *staurophorum* and their sexual progeny.

Specimens named *P*. *staurophorum* isolated from Guam (clone HK212; *P*. aff. *staurophorum* in Table 5) and examined by [32] are closer to Gregory's description in density of their striae, but they have much wider valves (Fig 15) than those from the original Scottish material, our clones, or other reports of this species in major monographs (Table 5). The clone is genetically distant from 18S and *rbc*L sequences of Canadian Atlantic clones of *P*. *staurophorum* (Fig 10). It also did not show any sexual interaction with our *P*. *staurophorum* or a new species discussed below (Table 3). All this suggests that clone HK212 [32] and some other diatoms referred to as *P*. *staurophorum* in some well known taxonomic monographs [59, 66, 78, 82] may represent new species belonging to a *P*. *staurophorum* species-complex, rather than all representing one, original concept of this species [61].

#### *Plagiogramma tsawwassen* Kaczmarska and B. S. Gray Jr., species nova (Fig 16)

**Fig 16:**
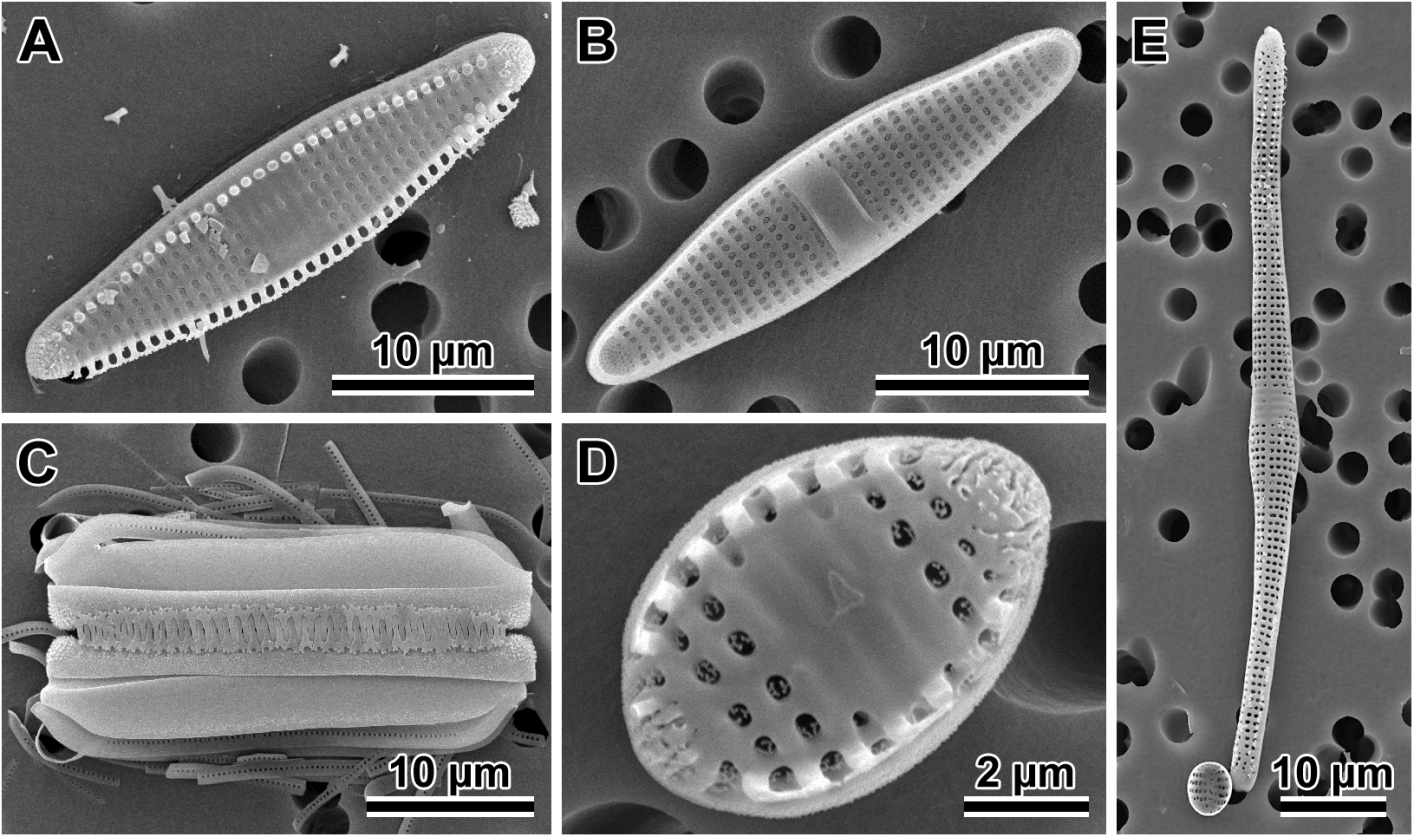
Plagiogramma tsawwassen. **(**A) External and (B) internal view of a typical vegetative valve face representing material from holotype preparation No. B 40 0042017 at BGBM on SEM stub 275–5 from clone Van5:1. (C) Girdle view of two sibling valves showing colony formation from the same preparation. (D) External view of a valve face of a very small specimen from Van5:5 clonal culture after nearly 6 years in culture. (E) External view of a sexual progeny from mating Van5:5 with Van5:6 showing some of the common imperfections in early post-sexual valves; here, asymmetry of the valve and proliferation of spines over the entire valve face.

Holotype: DMF SEM stub 275–5 deposited as BGBM preparation No. B 40 0042017 and illustrated in Fig 16A; in culture as clone Van5:1; fixed, non-cleaned material from that cultured clone as BGBM preparation B 40 0042018. Differential interference contrast (DIC) light photomicrographs of the valves are shown in S3E and S3F Fig. Sequence fragments of *rbc*L, SSU rDNA gene and the ITS region are deposited in GenBank and BOLD System (accession numbers in Table 1).

Type locality: intertidal mudflat at the ferry terminal at Tsawwassen, near Vancouver, British Columbia, Canada; geographic coordinates given in Table 1.

Description: Chain forming diatom. Depending on the apical valve length, the valve outline lanceolate (Fig 16A and 16B) or elliptical (Fig 16D) with a flat valve face and a distinct mantle. Near-natural (those examined soon after establishing the clone in 2010–2011) and post-sexual valves 9–87 μm long and 5–8 μm wide; large apical pore fields slightly elevated above the valve face surface carry small spines and papillae (Fig 16A, 16C and 16D). Mantle and sometimes valve face granulose (Fig 16C). Branched marginal spines (Fig 16A) located on interstriae at the valve face-mantle junction (Fig 16C). On post-sexual valves, spines dispersed throughout the valve face (Fig 16E). The central portion of the valve face forms hyaline fascia. Striae uniseriate, continuing onto the mantle, ending just below the spines, 13–18 in 10 μm, may appear uninterrupted by the sternum, but the sternum is clearly present during valve morphogenesis. Striae areolae large, covered by reticulate rota, 12–23 in 10 μm (Fig 16B and 16D). More morphometric data is found in Table 2.

Internally, valve surface bisected by two costae, one at each end of the central fascia. The fascia coincides with the area between the costae, and may form one ovoid or two side-by-side, circular divots (Fig 16B). Areolate striae separated by strongly thick interstriae, rimoportulae are absent. Apical pore-fields located in a semi-circular, apical depression clearly separated from the row of striae. There are several open copulae per theca (Fig 16C). Valvocopulae unperforated, all other copulae narrower and with one row of pores. Valve width and number of striae are the least variable characters of valve morphology among those examined here. The relationship between valve width and striae density in valves 9–88 μm long is illustrated in Fig 15 as characters likely to be most useful in species recognition. Differential interference contrast (DIC) light photomicrographs of the valves are shown in S3E and S3F Fig.

Diagnosis: Morphological signature: narrow, nearly indistinct sternum, striae fine, 13–18 in 10 μm, uniseriate, consisting of 12–23 areolae in 10 μm with reticulate rota.

Molecular signature: consists of two nuclear encoded DNA fragments, a conserved section of the SSU rRNA gene, the ITS region, and a plastidal DNA fragment from the *rbc*L gene; accession numbers in Table 1.

Comparison to similar congeners: *Plagiogramma* is also a relatively small genus with 50–60 species attributed to it, depending on the source. We examined the literature record of about 40 of the most commonly reported species for which we were able to obtain both descriptions and image data. Because the valve view for some species has not yet been determined (e.g., *P*. *jamaicense* Grev., *P*. *ornatum* Grev.) only a limited comparison of morphology for such species is possible.

Species so examined may be divided into two categories: those without internal costae (e.g., *P*. *seychellarum* Grun. and *P*. *tessellatum*) and those with costae, which are nearly all other members of the genus. Furthermore, among the taxa with internal costae, two groups are known: those with two, and those with more than two internal costae. Thus far two costae is the most common condition in the genus (26 species of 41 examined here in detail), and our new species falls within this category. About a dozen species have lanceolate-to-elliptical valves with two internal costae in the mid-valve region (Table 5). Other two-costa species can readily be differentiated from our new species due to their undulated valve outlines, mid-valve swelling or capitate apices.

While clearly similar in valve structure to *P*. *staurophorum*, *P*. *tsawwassen* valves show finer striation (Fig 15). There are only a few finely striated species in this genus (Table 5). Cleve [87] described *P*. *tenuistriatum* from the warm waters of Labuan, Malaysia, collected during the Vega Expedition, and emphasized that striae of this diatom were finer than any other species known at the time ([87] p. 498, Plate 37. fig. 63). This species has rarely been reported with associated metrics and image support, with the notable exception of [59] from Beaufort, North Carolina, USA. However, neither the shape of the fascia nor the metrics (calculated from [59], fig. 28; valve 20.8 μm long, 4.9 μm wide, 23–24 striae in 10 μm and 28 pores in 10 μm) of the North Carolina specimen conforms to Cleve’s species delineation and illustration of the original specimen.

Our smaller valves are similar to Cleve's specimens in valve length and density of striae. However, the valve outline of our larger specimens, comparable to those shown by Cleve, is not elliptical, but narrowly lanceolate; altogether the valve proportions are different. Specimens of *Plagiogramma* aff. *staurophorum* (clone HK212; [32]), are similar to *P*. *tenuistriatum* in valve outline, but not in valve width or striae count (Table 5).

Distribution: This species is known from three different sites on Vancouver Island, BC, Canada: Tsawwassen is located on the coast of the Salish Sea between the BC mainland and Vancouver Island, while Ucluelet and Tofino are located farther north on the open Pacific shore, some hundreds of kilometres from Tsawwassen. Clone HK324, isolated from Potlatch State Park in Washington State, USA, approximately 300 km south of Tsawwassen [32], shares metrics (valve 15–26 μm long, 6.6–7.7 μm wide, 13–14 striae and 13–18 pores in 10 μm) and genetic characteristics with our new species (Fig 15), and probably is conspecific.

## Discussion

### Sexual reproduction and life history

Among the diatoms, araphid species are some of the least investigated, with only about a dozen of the known genera having any aspect of reproductive biology examined in any detail for any species. The interest in applying reproductive characters in phylogenetic studies of araphid diatoms has been slowly growing in the last decade [13, 14, 31, 88], because sex-related characters are relevant for species survival, and as such they are generally strongly conserved. This is why they are also often used to infer deep divergences in a wide range of organisms [89–91], including diatoms [2, 6–8, 24, 27, 92].

From the existing small body of knowledge, araphids emerge as a group with a wider range of variability in sexual processes employed when compared to the centrics, with thus far universal flagellated sperm involving oogamy, or to raphid pennates with non-flagellate motility of parents and iso- or anisogametangiogamy. In araphids, amphimixis includes oogamy with small, motile spermatia and large immotile eggs (*Rhabdonema* and *Grammatophora*; [18, 27, 93, 94]), physiological and behavioural anisogamy of more or less equal size gametes, each with sex-specific behaviour and morphology [19–21, 95], and strict isogamy (*Meridion circulare*, at least in some reports; [95, 96]). The most commonly reported thus far is anisogamy: behavioural (exemplified in species such as *Licmophora communis* [97]), and behavioural and morphological, exemplified by *Tabularia fasciculata* [20] or *Ulnaria ulna* [23].

Similarly, sexual reproduction in our "basal" araphid species, the first ever examined, is behaviourally and physiologically anisogamous involving stationary females and motile males. In addition, similar to such genera as *Tabularia*, the mating system of some of our plagiogrammacean species is rather complex. For example, in addition to unisexual clones present in all three species, in some of them (*P*. *tsawwassen*) the phenotypic expression of sex also includes polysexual and intraclonal auxosporulation. This may reflect transition in the sex-identity structure within the species populations, from ancestral generally homothallic and self-fertile centrics to more derived "core" araphids and raphid diatoms where heterothallism becomes more common.

Thus far, pseudopodial motility of male gametes is a reproductive character of sex cells that seems to be known only among araphid species (both basal and core). Sluggish in some clones, but much more vigorous in others, this cell motility is driven by extrusion and retraction of protoplastic protrusions in the form of broad and slow, or fast whip-like pseudopodia. In our three species, motile cells may be both the secondary spermatocytes (uninucleate male cells) and male gametes (binucleate male cells). Even secondary spermatocytes may fuse with female gametes, although syngamy has to be delayed in such cells. Female secondary oocytes and gametes are capable of limited motility. They have been observed to vacate the gametangial theca, and then jitter about at the rim of the opened theca with most of the gamete surface exposed to male gamete interaction. A stationary behaviour of one of the compatible gametes is an advantage when the search for a partner is random [98]. Evolution of the raphe and motility of the entire raphid diatom cell (including gametangia-to-be) likely affords an adaptive advantage of greater protection for sex cells while searching for a compatible partner.

The evolutionary advantage of the loss of a flagellum in male gametes by pennates, compared to polar centrics, has long been difficult to understand. This would be particularly difficult in reference to generally immotile araphid pennates because it is logical to expect that immobility of gametes may negatively impact fertilization success for detectable sexually compatible but physically too distant gametangia. However, if indeed the last common ancestors of pennates had motile secondary spermatocytes (as we observe in our "basal" araphid species), then the loss of a flagellum on male gametes might have been less taxing than anticipated. Furthermore, a generally larger cell and an eventually binucleate cell, such as a secondary spermatocyte and eventually male gamete, may be considered as having a greater energy reservoir available for motility, and a "back-up" gametic nucleus to further increase probability of fertilization, should one of the nuclei become suboptimal. It is unknown at this time whether the loss of flagella resulted in secondary spermatocytes taking up a gametic function due to the earlier loss of flagella, or vice-versa, whether the secondary meiocytes taking up a gametic function made the flagellated sperm obsolete. Which of the two evolutionary pathways evolved earlier will soon be testable by examination of the genes responsible for the flagellar apparatus in polar centrics and araphid diatoms.

Pyknosis of supernumerary nuclei resulting from Meiosis II is delayed in our species. This leads to 3–4 equally sized nuclei in young and even already expanding auxospores. We are not aware of such auxospores in other araphids although quadri-nucleate zygotes had been reported in some raphid diatoms [99].

### Auxospore ontogeny and wall structure

Auxospore ontogeny and wall fine structure is known for just a few genera of araphids: *Rhabdonema* spp. [2, 18], *Gephyria* [36], *Grammatophora* [14], *Pseudostriatella* [13], and two species of *Tabularia* [15, 100]. Although there are variations in detail, a few consistent characters emerge here as well. In most of these species, auxospore walls consist of incunabular, scaly elements in their earliest, spherical stages of development, similar to other polar diatoms, both centric and raphid. Growing, anisodiametrically expanding, auxospores lay down circumequatorially transverse perizonia (TP) to facilitate elongation. At least in one of our species, this is accomplished not by sequentially adding bands to each apex (as reported in many pennate diatom auxospores), but rather depositing all/most of the bands all at once and then pushing them out like a collapsible cup as the auxospore elongates. We observe that the TP bands are more slanted near apices in our species than is generally seen in pennates, which is a character similar to some polar centric auxospores (e.g., *Chaetoceros* spp., *Biddulphia* spp.; [2]) and some "core" araphids (*Gephyria*, *Pseudostriatella*; [13, 36]). The TP is made up of bands that are open in all the species examined here. In comparison, in both polar centrics and in pennates, transverse perizonial bands may or may not be open; von Stosch ([2]; e.g., p. 137, 140) gives several examples of each type. In two araphid species (*Grammatophora marina* and *Pseudostriatella oceanica*), some transverse perizonial bands are closed rather than open, similar to those seen in some polar centric auxospores. Among diatoms thus far examined, polar centric diatoms are more likely to have more closed transverse perizonial bands than open ones, while polar pennates are more likely to have them open than closed.

Longitudinal perizonial bands (*sensu* [100]; similar to TP of polar centrics) are present in some araphid diatoms. They were absent in all three of the species examined here. In our species, a relatively lightly silicified structure perforated by simple, small pores (rather than areolae) is an initial epivalve rather than longitudinal perizonium [4]. Identification of this structure as an initial epivalve is demonstrated here when its formation is preceded by an acytokinetic mitosis, as in most documented geneses of diatom vegetative valves. Some of the similar structures reported earlier in [15, 101, 102] and others may also represent an initial epivalve rather than a longitudinal perizonium. The initial valvocopula was ornamented in all three species examined here, in contrast to valve copulae of vegetative valves. It also may be closed in *D*. *acutumontgo* and in *P*. *tsawwassen*, again in contrast to typical vegetative valves of any other species examined. In our species, we did not observe the longitudinal structures (longitudinal perizonium as shown in [2, 100]; sprawling, or irregular structures) and scales that can cover the auxospore protoplast when the TP bands fall short of the auxospore circumference in some species.

In summary, sexual reproduction of the first three members of the basal araphid clade ever investigated and shown here, demonstrate many characters reported also in "core araphids". These are: cis-type allogamic mating behaviour, male gametes vigorously motile due to their pseudopodial activity, scales in the cell wall of the youngest stages of auxospore development, and open, bidirectional elongation of transverse perizonial bands in tubular auxospores. These characters were likely present in the last common ancestors of the "basal" araphids and the diatom clade containing the "core" araphids and raphid pennates.

The characters thus far uniquely present among our plagiogrammaceans, but not reported from other araphid pennates are: 1) the fusion between secondary meiocytes, in some cases before completion of Meiosis II in males; 2) many or all transverse perizonial bands produced all together or in quick succession rather than added one at a time at the auxospore apex as it elongates; and 3) three to four nucleated young auxospores which retain two gametic nuclei from each compatible partner after the beginning of auxospore expansion. An initial epivalve, similar in morphology to what in some earlier publications was interpreted as “longitudinal” perizonium, found here, is possibly more widespread among pennates than thus far appreciated. However, because ours is the first report on sexual auxosporulation in basal araphid diatoms, it remains to be confirmed whether these characters are indeed characteristic of, and restricted to, basal araphid taxa.

### Taxa examined

Three syngens were discovered among the clones examined: one from *Dimeregramm*a and two from *Plagiogramma*. Among these, two new species are proposed (*D*. *acutumontgo* and *P*. *tsawwassen*). One taxon represented by two clones was considered morphologically unidentifiable because of their extraordinary small cell-size. The DNA sequences available for these two clones do not BLAST to any identified vouchered specimens. The identity of our *P*. *staurophorum* clones is verified by their comparison to Gregory’s original material curated at the British Museum. Our clones are provided with molecular signatures in order to aid consistent recognition of this taxon and its typical specimens in future research.

Our results support the notion that species level diversity of diatoms currently recognized within these two genera is far from complete. Of the two *Dimeregramma* species we found, one was proven new to science even though it appears to have a wide distribution in relatively well-investigated boreal temperate seas. Between two syngens of *Plagiogramma*, one species, *P*. *tsawwassen*, is new to science, but it differs only subtly from the morphological delineation of *P*. *staurophorum* (Gregory) Heiberg and may be relatively easily confused with it. The two species do not interbreed and are genetically distinct. Furthermore, yet another entity named *P*. *staurophorum* was reported from Guam [32] which fits the specific delineation of *P*. *staurophorum* in all but one character; the valve width is much greater. The clone representing this species (HK212; [32]) does not interbreed with either of the most sexually competent clones of our *P*. *staurophorum*, under our conditions of sexual induction. This is not surprising given the degree of genetic divergence between them. The diatom represented by HK212 may also be a species new to science. The metric characters also separate this clone from specimens of *P*. *staurophorum* found in the original material, from which our specimens are morphologically indistinguishable. Widespread cryptic or semi-cryptic diversity has been reported in numerous earlier studies applying morphological, molecular and biological means of species delineation. This theme appears to be recurring in recent diatom research [9, 103–105]. Thus, our results add support to the contention that our knowledge of diatom species level diversity is still insufficient [106].

Both genera (*Dimeregramma* and *Plagiogramma*) belong to the family Plagiogrammaceae. Four-gene phylogeny and intrafamilial relationships between its generic members has recently been investigated by Li et al. [32]. These authors recovered Plagiogrammaceae as a member of the “basal” araphids, as did others before them. However, the integrity of the genus *Dimeregramma* was questioned in that study. Despite relatively consistent morphological differences in their valve microarchitecture, the genera *Plagiogramma* (valves with internal costae) and *Dimeregramma* (valves with no internal costae but pronounced interstriae and “flappy” marginal spines) are paraphyletic in molecular phylogenies. Similar to [32], our phylogenetic analysis (based on partial sequences from nuclear encoded SSU and plastidal *rbc*L) also shows that at least one clone (SZCZP43) with a *Dimeregramma* valve morphology joins the clade with our species of *Plagiogramma* and two other genera. Taken altogether, our conclusions and those of [32] converge to the same point: both genera require serious taxonomic reappraisal.

## Supporting information

S1 Fig Maximum likelihood phylogenies of 22 Plagiogrammaceae strains from this study, for three marker genes. **(**A) SSU; (B) rDNA; (C) *rbc*L and ITS. Trees inferred using RAxML v. 8.2.0. Outgroups are *Delphineis* CCMP 1095, *Asterionellopsis* cf. *glacialis* CCMP139 and *Asteroplanus* aff. *karianus* CCMP 1717 (reported as *A*. *socialis* ECT3920 in [32]). Bootstrap values > 500 (out of 1000) are shown as percentages above or below the node. Scale bar shows number of substitutions per position.
(TIF)

S2 Fig Bayesian phylogeny of Plagiogrammaceae strains. (A) Three concatenated markers, SSU rDNA, ITS, and *rbc*L. (B) Two concatenated markers, SSU rDNA and *rbc*L. Outgroups are: *Asterionellopsis* cf. *glacialis* CCMP139 and *Asteroplanus* aff. *karianus* CCMP 1717 (reported as *A*. *socialis* ECT3920 in [32]), and *Delphineis* sp. CCMP 1095; taxonomic justification for species names in [9]. Values above or below nodes show Bayesian posterior probabilities. Scale bar shows number of substitutions per position. Names of strains from this study are given in full in Table 1.
(TIF)

S3 Fig Differential interference contrast (DIC) light microscopy of *D*. *acutumontgo*, *P*. *staurophorum* and *P*. *tsawwassen*. (A and B) *D*. *acutumontgo* from StA:5 culture material archived in 2015. (A) Lanceolate valve. (B) More elliptical valve. (C and D) *P*. *staurophorum* from StA:8 culture material archived in 2015. (C) Lanceolate valve. (D) More elliptical valve. (E and F) *P*. *tsawwassen* from Van5:1 culture material archived in 2015. (E) Lanceolate valve. (F) More irregular elliptical valve (right) with mantle view of pair of sibling valves (left) with obvious fascia.
(TIF)
