# Supplementary figures and images for: Sexual reproduction in plagiogrammacean diatoms: First insights into the early pennates
Source: PLoS One. 2017 Aug 16;12(8):e0181413. doi: 10.1371/journal.pone.0181413 (PMC5558960; doi:10.1371/journal.pone.0181413)

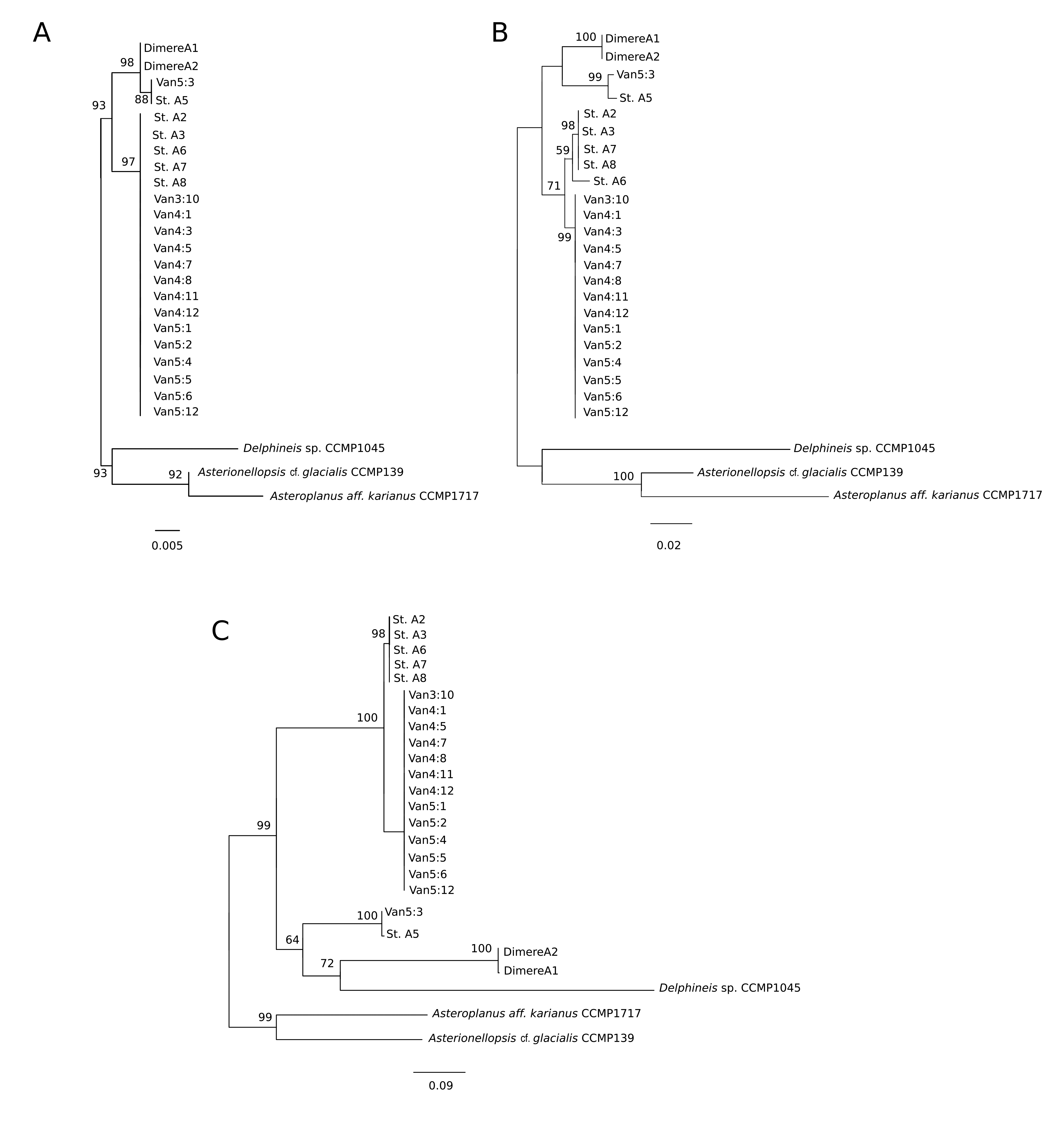

Supplement: S1 Fig — (A) SSU; (B) rDNA; (C) rbcL and ITS. Trees inferred using RAxML v. 8.2.0. Outgroups are Delphineis CCMP 1095, Asterionellopsis cf. glacialis CCMP139 and Asteroplanus aff. karianus CCMP 1717 (reported as A. socialis ECT3920 in [32]). Bootstrap values > 500 (out of 1000) are shown as percentages above or below the node. Scale bar shows number of substitutions per position. (TIF) [file pone.0181413.s001.tif]

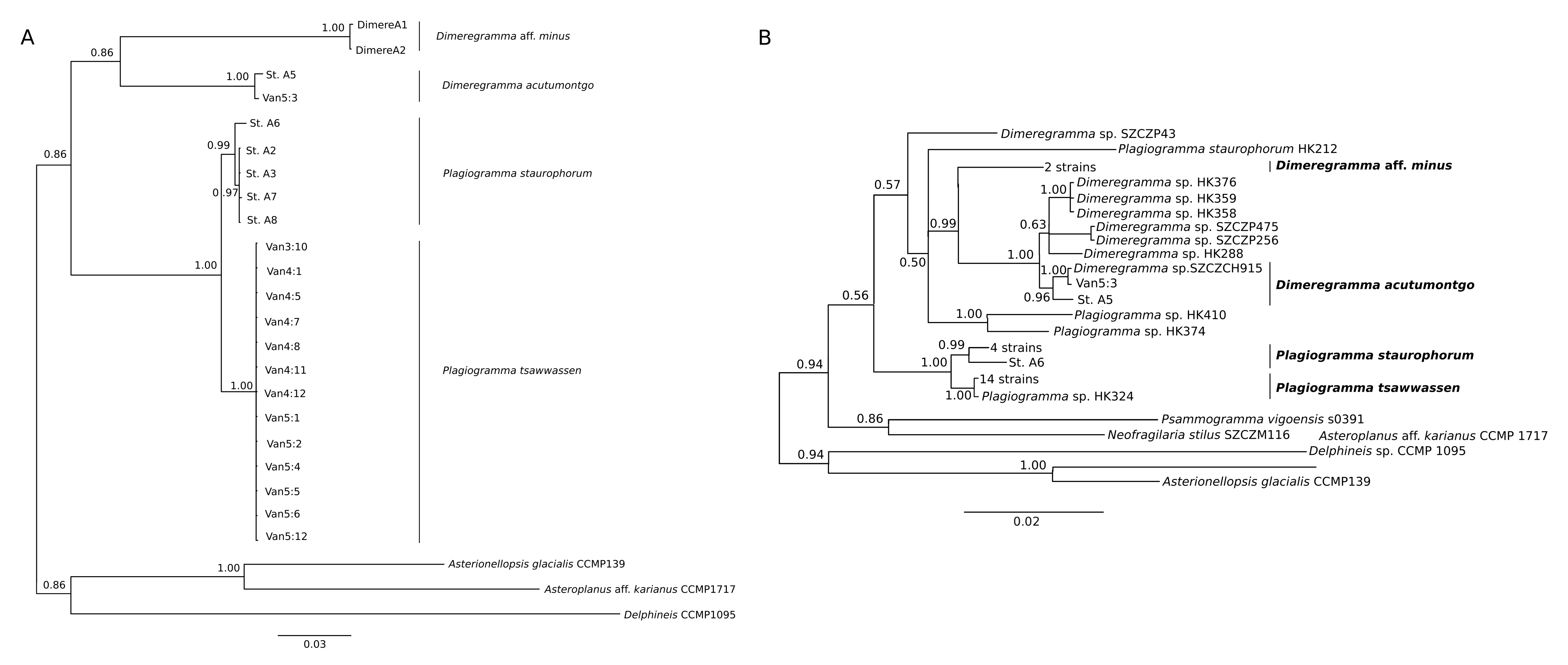

Supplement: S2 Fig — (A) Three concatenated markers, SSU rDNA, ITS, and rbcL. (B) Two concatenated markers, SSU rDNA and rbcL. Outgroups are: Asterionellopsis cf. glacialis CCMP139 and Asteroplanus aff. karianus CCMP 1717 (reported as A. socialis ECT3920 in [32]), and Delphineis sp. CCMP 1095; taxonomic justification for species names in [9]. Values above or below nodes show Bayesian posterior probabilities. Scale bar shows number of substitutions per position. Names of strains from this study are given in full in Table 1. (TIF) [file pone.0181413.s002.tif]

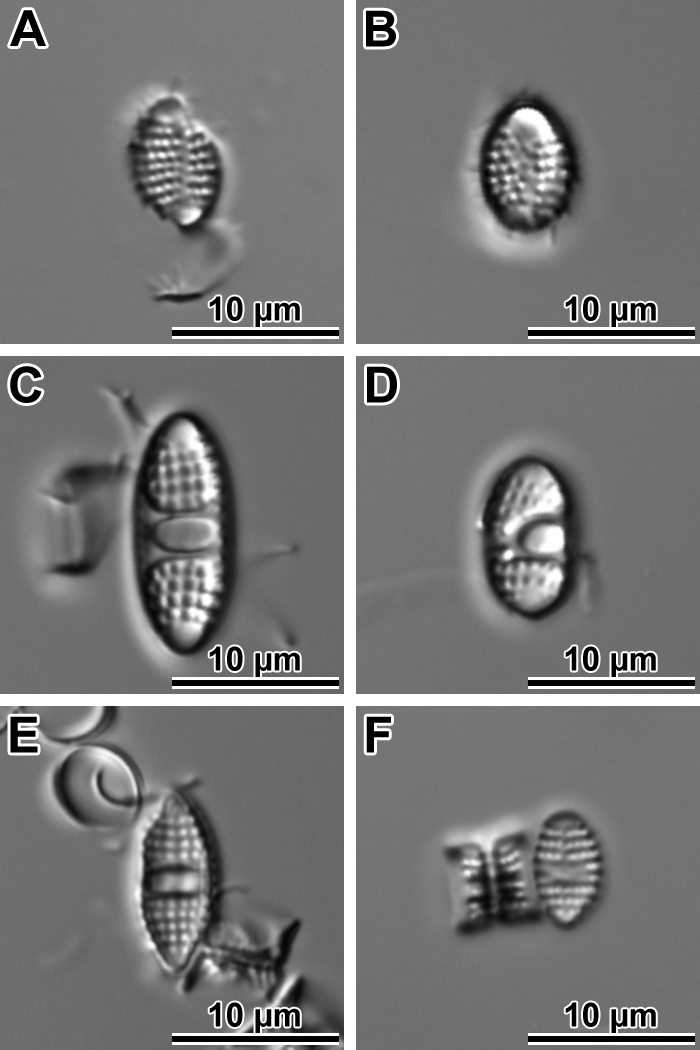

Supplement: S3 Fig — (A and B) D. acutumontgo from StA:5 culture material archived in 2015. (A) Lanceolate valve. (B) More elliptical valve. (C and D) P. staurophorum from StA:8 culture material archived in 2015. (C) Lanceolate valve. (D) More elliptical valve. (E and F) P. tsawwassen from Van5:1 culture material archived in 2015. (E) Lanceolate valve. (F) More irregular elliptical valve (right) with mantle view of pair of sibling valves (left) with obvious fascia. (TIF) [file pone.0181413.s003.tif]
